# Advanced analytics and artificial intelligence in gastrointestinal cancer: a systematic review of radiomics predicting response to treatment

**DOI:** 10.1007/s00259-020-05142-w

**Published:** 2020-12-16

**Authors:** Nina J. Wesdorp, Tessa Hellingman, Elise P. Jansma, Jan-Hein T. M. van Waesberghe, Ronald Boellaard, Cornelis J. A. Punt, Joost Huiskens, Geert Kazemier

**Affiliations:** 1 Department of Surgery, Cancer Center Amsterdam, Amsterdam University Medical Centers, Vrije Universiteit, Amsterdam, The Netherlands; 2 Department of Epidemiology and Biostatistics, Amsterdam University Medical Centers, Vrije Universiteit, Amsterdam, The Netherlands; 3 Department of Radiology and Molecular Imaging, Cancer Center Amsterdam, Amsterdam University Medical Centers, Vrije Universiteit, Amsterdam, The Netherlands; 4 Department of Radiology and Nuclear Medicine, Cancer Center Amsterdam, Amsterdam University Medical Centers, Vrije Universiteit, Amsterdam, The Netherlands; 5 Department of Epidemiology, Julius Center for Health Sciences and Primary Care, University Medical Center Utrecht, Utrecht, The Netherlands; 6 SAS Institute B.V, Huizen, The Netherlands

**Keywords:** Gastrointestinal cancer, Advanced analytics, Artificial intelligence, Radiomics, Diagnostic imaging, Treatment response

## Abstract

**Purpose:**

Advanced medical image analytics is increasingly used to predict clinical outcome in patients diagnosed with gastrointestinal tumors. This review provides an overview on the value of radiomics in predicting response to treatment in patients with gastrointestinal tumors.

**Methods:**

A systematic review was conducted, according to PRISMA guidelines. The protocol was prospectively registered (PROSPERO: *CRD42019128408*). PubMed, Embase, and Cochrane databases were searched. Original studies reporting on the value of radiomics in predicting response to treatment in patients with a gastrointestinal tumor were included. A narrative synthesis of results was conducted. Results were stratified by tumor type. Quality assessment of included studies was performed, according to the radiomics quality score.

**Results:**

The comprehensive literature search identified 1360 unique studies, of which 60 articles were included for analysis. In 37 studies, radiomics models and individual radiomic features showed good predictive performance for response to treatment (area under the curve or accuracy > 0.75). Various strategies to construct predictive models were used. Internal validation of predictive models was often performed, while the majority of studies lacked external validation. None of the studies reported predictive models implemented in clinical practice.

**Conclusion:**

Radiomics is increasingly used to predict response to treatment in patients suffering from gastrointestinal cancer. This review demonstrates its great potential to help predict response to treatment and improve patient selection and early adjustment of treatment strategy in a non-invasive manner.

**Supplementary Information:**

The online version contains supplementary material available at 10.1007/s00259-020-05142-w.

## Introduction

Gastrointestinal (GI) cancer is one of the leading causes of cancer related deaths worldwide, resulting in approximately 2.8 million deaths annually [1]. To improve survival in these patients, local and systemic treatment strategies are often combined. Multimodality treatment can include resection, thermal ablation, radiotherapy, transarterial embolization, and systemic therapy [2–5]. Timing and intent of systemic therapy are subject to tumor stage and pathology of the specimen. Systemic treatment often has a palliative intent. However, systemic therapy or chemoradiotherapy can also be used as neoadjuvant treatment for downsizing the tumor to allow local treatment with curative intent [3, 4]. Given the possible adverse effects of treatment, selection and monitoring of patients are crucial for optimal treatment results [6, 7].

Over the past decades, clinical oncology is shifting more and more from “one therapy fits all” to “personalized cancer treatment”. Advancements in technology and increased knowledge about the underlying tumor biology contributed to this rise of personalized cancer care. For example, new targeted therapies attack or mimic specific molecules and pathways [8]. However, it remains challenging to select patients likely to benefit from treatment strategies as response can vary considerably between patients [2, 9]. Prediction of response to treatment could therefore lead to improved personalized treatment.

To predict response to treatment, predictive models with novel prognostic variables have emerged. Over the past decade, research using advanced analytics, like radiomics, has expanded substantially. Radiomics is an advanced method to extract imaging features and thereby quantify tumor phenotype from medical images [10]. With use of radiomics, more information can be obtained from a single medical image, since hundreds of imaging features can be extracted and analyzed. Radiomic image features are broadly grouped into morphological (size and shape) features, intensity-based features describing the distribution of voxel intensities (e.g., Hounsfield units), textural features describing the relationships between voxel values, and filter transformations [11].

Radiomics allows objective assessment of clinically relevant features, such as features depicting tumor heterogeneity as the human eye is not able to quantify tumor heterogeneity in an objective manner [10, 11]. Radiomics may therefore contribute to more objective and accurate response evaluations. Moreover, these imaging features can be used in predictive modeling in combination with other types of data [10–12]. Predicting response with radiomics could lead to selection of the most effective treatment based on patient- and tumor-specific characteristics in a non-invasive manner. Many studies have shown promising results of the use of radiomics for predicting response to treatment in patients with various types of cancer [13–15]. However, the predictive performance of radiomics in patients with gastrointestinal tumors is unclear. We therefore conducted a systematic review on the value of radiomics in predicting response to treatment in patients diagnosed with gastrointestinal tumors.

## Methods

### Search strategy

This study was performed according to the Preferred Reporting Items for Systematic Reviews and Meta-Analyses (PRISMA) guidelines [16]. A comprehensive literature search was conducted by a qualified librarian specialized in systematic reviews (EPJ). PubMed, Embase, and Cochrane electronic databases were searched from database inception until 12 December 2019. Synonyms for the following topics were reported as MeSH terms or keywords: gastrointestinal cancer, advanced analytics, and tumor response. The complete search strategy is listed in the Supplementary. The protocol of this systematic review was prospectively registered with PROSPERO (*CRD42019128408*) [17].

### Study selection

Two members of the research team screened and selected studies from the literature search independently (NJW and TH). Studies describing advanced analytics (i.e., radiomics or textural analysis) for prediction of tumor response to treatment in patients with a gastrointestinal tumor were included for analysis. Gastrointestinal tumors encompassed esophageal, gastric, (small) intestinal, colorectal, hepatic, pancreatic, and gallbladder cancer. Imaging modalities included (contrast-enhanced) computed tomography (CT), magnetic resonance imaging (MRI), and positron emission tomography (PET). Original studies, such as observational cohort studies and clinical trials, were selected. Other publication types, like reviews, meta-analysis, case reports, and conference abstracts, were excluded from analysis. Reasons for exclusion were stated, and disagreement was resolved by re-evaluation and discussion.

### Data collection and analysis

Data were extracted by author NJW and checked by author TH, using a pre-defined data extraction form. The following data were collected: study population, treatment strategy, timing and modality of diagnostic imaging for feature extraction, response assessment, number of analyzed and selected (radiomic) features, and best predictive performance, including discriminatory power and accuracy of (validated) models. If multiple individual features were assessed, the best predictive feature was reported. In the case that more than 15 parameters were selected, the parameters were not specified. Instead, a reference to the published paper was made. The primary outcome of interest was the predictive performance of radiomic features or models, reported as area under the receiver operating curve (AUC) and/or accuracy. A narrative synthesis of results was conducted, stratified by tumor type.

### Quality assessment

Risk of bias and methodological quality of included studies were assessed independently by both researchers (NW and TH), according to the radiomics quality score (RQS). The RQS was introduced to provide standardized evaluation criteria and reporting guidelines for radiomics research to minimize bias and enhance the usefulness of prediction models [10]. Sixteen key components were assessed, resulting in a maximum score of 36 points. A higher score indicated higher quality. Discrepancies in assessment of quality were resolved by re-assessment and discussion.

## Results

**Fig. 1:**
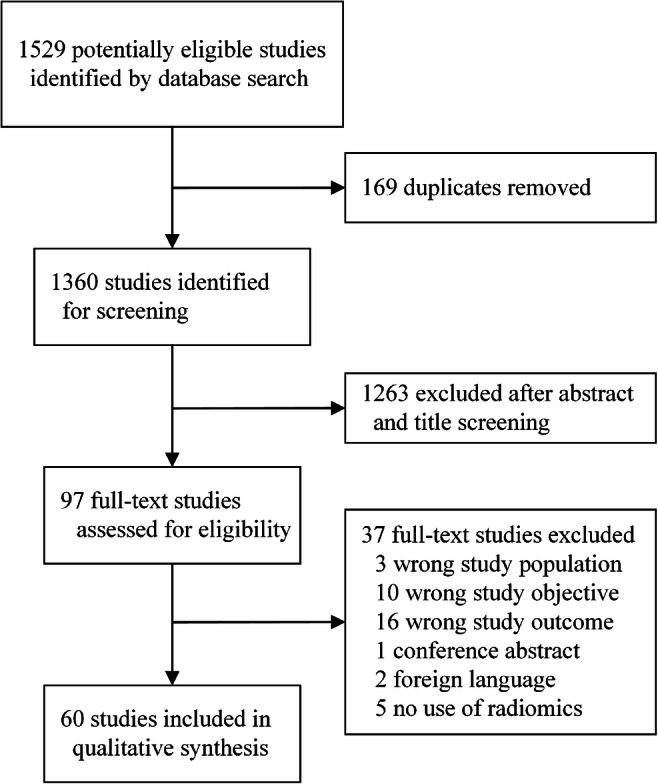
Flow diagram of study selection process

The comprehensive literature search identified 1360 unique studies, of which 1263 were excluded based on screening of title and abstract. Full texts of the remaining 97 studies were assessed for eligibility. Of these studies, 60 were included for analysis. Most common reason for exclusion is wrong study outcome, followed by wrong study objective (Fig. 1).

### Esophageal cancer

The predictive value of radiomics in patients with esophageal cancer was analyzed in 13/60 (21.7%) studies included for analysis. In the majority of these studies, complete pathological response was assessed after chemoradiotherapy. Radiomic features are most frequently derived from pre-treatment PET imaging (Supplementary, Table 1).

Radiomic features were combined with clinical parameters to construct a predictive model in 6/13 studies [18–23]. These prediction models resulted in high-performance levels with good discriminating power (AUC 0.69–0.92). Radiomics-based models and individual radiomic features often outperformed conventional metrics, such as standardized uptake values (SUV) measurements (AUC 0.50–0.60) [19, 21, 24, 25].

Performance of various methods to construct predictive models was analyzed [20, 21, 26–29]. Zhang et al. observed that the combination of clinical, conventional PET and radiomic PET features in a support vector machine (SVM) model achieved highest accuracy in predicting complete pathologic response, while Ypsilantis et al. showed that a convolutional neural network (CNN) outperformed machine learning classifiers, including SVM, logistic regression model (LR), random forest (RF), and gradient boosting [21, 28]. Two studies from Hou et al. compared various prediction models [26, 27]. An artificial neural network (ANN) and a SVM were constructed, based on CT and MRI features, respectively. Both models showed high accuracy in predicting response to treatment after external validation. No statistically significant difference was observed in the predictive performance of the ANN and SVM models after internal validation, implying that the choice of the models was not of substantial importance. Although most studies in patients with esophageal cancer showed radiomics to be predictive for response to treatment, one study showed no association between pre- or post-treatment radiomic features and response [30].

### Gastric and gastroesophageal cancer

In total, 6/60 (10%) included studies focused on patients with gastroesophageal cancer, including analysis of abdominal cavity metastases of gastric cancer and gastroesophageal liver metastases [31, 32]. In the latter, a lesion-based analysis on 196 metastases was performed [31]. Radiomic features were derived from CT imaging. In the majority of studies, response to chemotherapy is assessed, according to pathological regression grading and volumetric criteria (Supplementary, Table 1).

Two studies combined radiomic features with clinical parameters [33, 34]. A three-point risk classification score was obtained to predict complete response in patients with gastroesophageal cancer after chemoradiotherapy. This risk model, consisting of one radiomic feature and one clinical feature, showed strong negative association with complete pathologic response, overall survival, and progression-free survival [34].

In patients with gastrointestinal stromal tumors treated with tyrosine kinase inhibitors, higher levels of four texture features were positively correlated with disease progression. The combination of these four features showed best discriminatory power in predicting disease progression (AUC 0.83) [33].

Radiomics models were often constructed with the use of machine learning classifiers, including ANN, RF, and k-nearest neighbor (KNN). These models showed good discriminatory power (AUC 0.72–0.79) and accuracy (0.79–0.82) in predicting response to chemotherapy or radiotherapy [31, 32, 35, 36]. Hou et al. showed similar predictive performance between ANN and KNN models after external validation (accuracy 0.82) [32]. Li et al. compared different combinations of feature selection and classifier methods. The best predictive performance was achieved by the combination of the filter-based linear discriminant feature selection method with the RF classifier (AUC 0.72) [35].

### Primary colorectal cancer

In the majority of included studies, the predictive value of radiomics was analyzed in patients diagnosed with colorectal cancer (CRC). A total of 27/60 (45%) studies described prediction of response to treatment in patients with primary CRC, in particular in patients with locally advanced rectal cancer (LARC) [37–63]. Pathologic response to chemoradiotherapy (CRT) is most frequently assessed, and radiomic features are predominantly derived from MRI (Supplementary, Table 1).

The methodology among studies varied considerably. The predictive value of individual radiomic features was analyzed in 10 studies, while radiomics-based prediction models were constructed in the remaining 17 studies. Different types of individual radiomic features were associated with complete pathologic response to CRT [37, 39–45, 47, 63]. The predictive value of entropy, kurtosis, skewness, and tumor volume were most frequently described [37, 40–44]. Two studies reported no significant association between PET texture features and pathologic response to CRT in patients with LARC [38, 46].

In 4/17 studies, radiomic features were combined with clinical parameters to construct predictive models [48, 50, 61, 62] Prediction models of the remaining studies were solely based on radiomic features [47, 49, 51–60, 63]. In 16/17 studies, the models were found predictive for pathologic response with good discriminative power (AUC 0.72–0.98) [47–52, 54–63]. The highest predictive performance was achieved by the model constructed in the study of Liu et al., which was based on a radiomics signature and tumor length (AUC 0.98) [62].

Radiomics models were often constructed with the use of machine learning classifiers, such as RF, SVM, ANN, and deep neural network (DNN) [48, 51–53, 56–58, 61]. In several studies, radiomics models were compared. Bibault et al. showed that a DNN outperformed the SVM model, created on the same features. Moreover, improved predictive performance was obtained compared to the LR model based on TNM staging [48]. Shayesteh et al. analyzed machine learning classifiers individually and together for response prediction and reported best predictive performance for the ensemble of machine learning models [57]. Two studies assessed the added value of radiomics models in addition to radiologists assessment. Where Horvat et al. observed that the radiomics model outperformed the qualitative assessment of clinical complete response by radiologists, Van Griethuysen et al. showed similar predictive performance in radiologists’ assessment of a subjective morphologic risk score [54, 60]. Even though the majority of studies reported good predictive performance of radiomics in patients with LARC, Hamerla et al. reported no predictive value of radiomics in response to treatment by a RF model, after results were corrected for imbalanced distribution (accuracy 50%) [53].

### Metastatic colorectal cancer

A total of 6/60 (10%) included studies analyzed the predictive value of radiomics in patients with metastatic CRC, of which five studies focused solely on patients with colorectal liver metastases [64–69]. In the majority of the studies, patients were treated with chemotherapy, and radiomic features were extracted from CT imaging before treatment. Response is assessed according to RECIST, pathological regression grading, volume criteria, and diameter change per lesion (Supplementary, Table 1).

Individual radiomic features were assessed in 5/6 studies, showing good discriminative power in three studies (AUC 0.74–0.81), while in two studies, no significant association was reported in multivariate analysis [65, 67]. Zhang et al. performed a lesion-based analysis on 193 metastases and found that features variance and angular second moment were predictive for response, while Ahn et al. found lower skewness and narrower standard deviation most predictive for response [64, 69]. Notable was that Van Helden et al. observed higher values of mean entropy in patients without response, defined as stable and progressive disease, while Beckers et al. observed a trend toward higher values of entropy in responders [65, 68]. Another study demonstrated that the change (delta) in entropy and uniformity was most predictive for pathologic response in univariate analysis, even though the potential predictive value did not remain in multivariate analysis [67].

A radiomics model was constructed by Creasy et al. predicting volumetric response with an average 20% prediction error. Clinical parameters were not combined with radiomic features in constructing the model, but the association between clinical parameters and response to treatment was individually assessed. Parameters associated with response were *KRAS* mutation status, age, systemic therapy regimen, and treatment strategy (*p* < 0.05) [66].

### Hepatic cellular carcinoma

The predictive value of radiomics in patients with hepatocellular carcinoma (HCC) was assessed in 4/60 (6.7%) studies [70–73]. Predominantly, radiomic features were derived from CT imaging in patients treated with transarterial chemoembolization. Tumor response is assessed according to RECIST, modified RECIST, and Response Evaluation Criteria in Cancer of the Liver (Supplementary, Table 1).

The predictive value of individual radiomic features was assessed in 3/4 studies. Park et al. performed a lesion-based analysis on 132 HCCs and found that several 2D and 3D texture features in arterial phase were predictive for complete response (AUC 0.59–0.72) [72]. Kloth et al. reported predictive value of texture features in both arterial and portal venous phase for complete response (AUC 0.74–0.80) [71]. Yu et al. found that lower pre-treatment entropy and post-treatment entropy and skewness were predictive for early response (AUC 0.65–0.76) in both phases [73].

Cozzi et al. constructed two models, comprising of radiomic and clinical features in patients treated with volumetric modulated arc therapy. In both LR models, a different single radiomic feature was selected to predict response, namely gray-level non-uniformity and energy, showing intermediate discriminative power for response (AUC 0.64 and 0.67) [70].

### Pancreatic cancer

In total, 4/60 (6.7%) included studies focused on patients with pancreatic cancer [74–77]. In the majority of the studies, radiomic features were extracted from CT imaging. Response after chemotherapy, chemoradiotherapy, or radiotherapy is assessed according to RECIST or pathologic grading of the resected specimen (Supplementary, Table 1).

The predictive value of individual radiomic features was analyzed in 3/4 studies. Clinical parameters were included in two of these studies to determine whether radiomic features could be considered independent prognostic factors in multivariate analysis [74, 77]. Borhani et al. found that higher values of mean positive pixel and chemotherapy regimen were associated with favorable pathologic response [74]. They also found that changes in kurtosis and skewness were correlated with biochemical response, defined as more than 50% decrease of CA19-9 levels [74]. Yoo et al. concluded that multiple texture and SUV features changed during treatment and were able to differentiate responders from non-responders [77]. In the study of Ciaravino et al., patients that underwent resection after downsizing neoadjuvant therapy were compared to patients with disease progression during neoadjuvant therapy. The results showed that kurtosis differed significantly before and after neoadjuvant therapy in the downstaged group. No significant changes between texture features were found in the disease progression group [75].

Nasief et al. constructed a machine learning model for early response prediction after neoadjuvant chemoradiotherapy. They constructed a Bayesian neural network (BNN) incorporating delta radiomic features with high discriminatory power (AUC 0.94) [76].

### Radiomics quality score

**Fig. 2:**
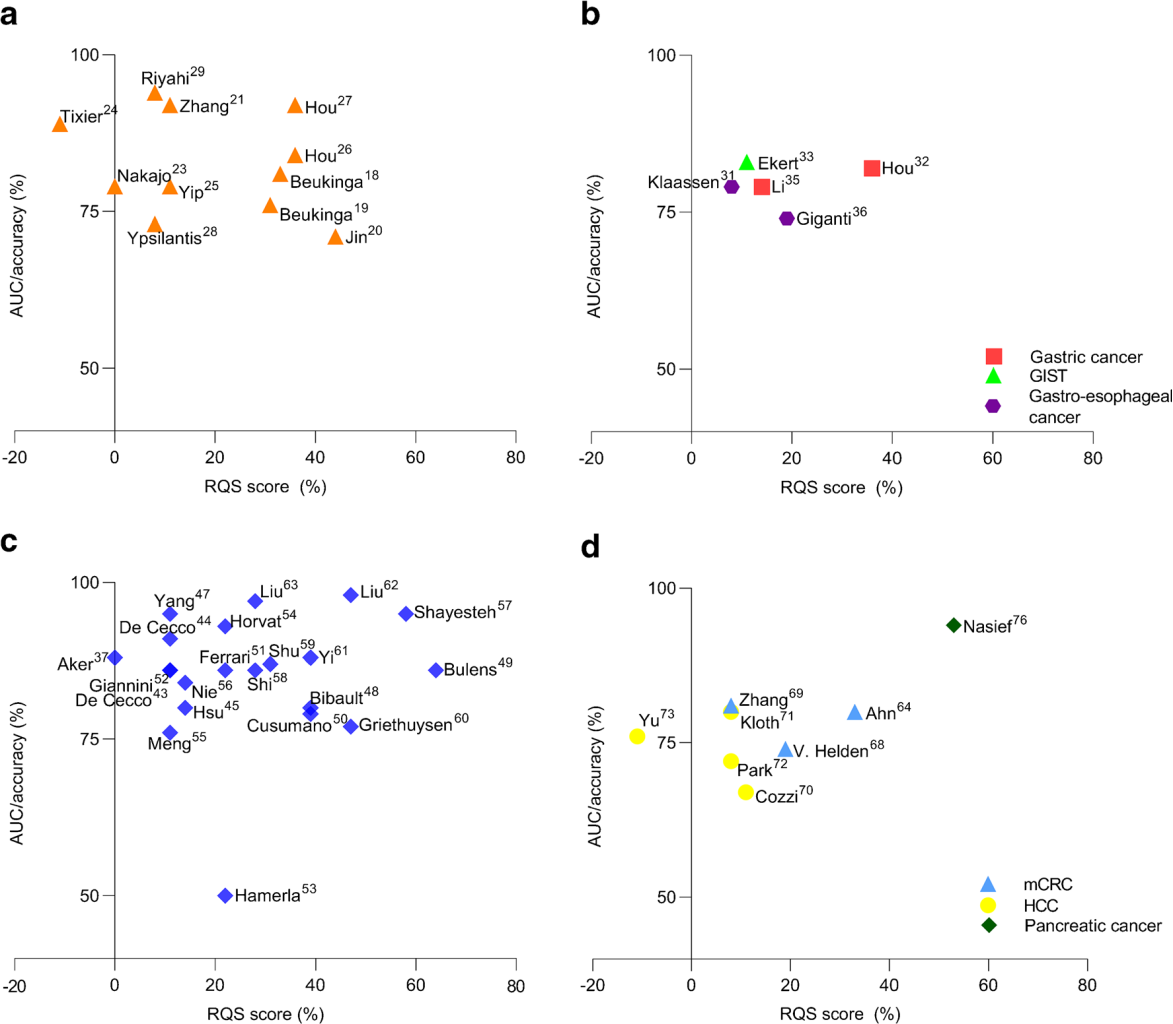
Overview of the radiomics quality scores (RQS) plotted against the best predictive performance of the radiomics models or features for different tumor types: (a) esophageal cancer, (b) gastric and gastroesophageal cancer, and gastrointestinal stromal tumors (GIST), (c) primary colorectal cancer, (d) metastatic colorectal cancer (mCRC), hepatic cellular carcinoma (HCC), pancreatic cancer. *X*-axis is the RQS score depicted as percentage of the maximum score (36 points = 100%). *Y*-axis is the best predictive performance of the radiomics features or models in terms of area under the curve (AUC) or accuracy

The radiomics quality scores (RQS) of included studies are shown in the Supplementary data, Table 2. The RQS ranged from − 4 to 23 out of 36 points (− 11–64%), with a median of 5 points (14%). No clear definition of high or low quality was formulated in the RQS guideline; however, only 19 studies scored over 30%. Different components for the quality and generalizability of prediction models were assessed, including feature robustness, overfitting of the model, external validation, and use of a prospective design. Total RQS scores are plotted against the best predictive performance of the radiomics models or features (Fig. 2). Studies with missing data on best predictive performance (accuracy or area under the curve) were excluded from these plots. No feature reduction or adjustment for multiple testing was performed in 14/60 (23%) of studies, which may have resulted in overfitting of models [22–24, 37, 39–44, 67, 73–75]. Study populations were relatively small compared to the number of parameters analyzed. A median of 65 patients [range: 8–235] was included per study, while a median of 72 parameters [range: 6–19,985] was assessed. Forty-five (75%) studies included less than 100 patients. In addition, studies often failed to maintain the “one in ten” rule of thumb, encompassing a minimum of ten patients for each predictive variable in the model (Supplementary, Table 1). Internal validation was performed by applying resampling methods in 24/60 (40%) of studies, such as bootstrapping and cross-validation. However, only 15/60 (25%) studies performed validation in an external dataset [20, 22, 26, 27, 32, 34, 49, 50, 57, 60–62, 64, 66, 76]. Five points were subtracted if studies lacked external validation, as this is one of the most important components for model generalizability. A prospective study design was conducted in only 7/60 (12%) of studies [43, 44, 49, 57, 59, 63, 77].

## Discussion

This review shows the potential of radiomics in predicting response to treatment in patients with GI cancer. High discriminatory power and accuracy of individual radiomic features and radiomics models were reported, in particular for rectal and esophageal cancer. In this review, the individual feature entropy, skewness and kurtosis were most frequently found predictive of response. However, methodology varied considerably among studies, particularly in feature extraction and selection, model construction, and validation. Results between studies were therefore difficult to compare. The majority of studies analyzed small study populations, potentially resulting in overfitting of models. Moreover, studies often lacked external validation, which affected the generalizability of the prediction models. To determine the added value of radiomics in clinical practice, direct effects of validated prediction models need therefore to be further clarified.

Features describing tumor heterogeneity were most frequently observed to be predictive of response. Entropy describes the pixel randomness and dissimilarity within a grayscale image; thus, higher values of entropy reflect a heterogeneous distribution of pixels [11]. Tumor heterogeneity has impact on clinical outcome and tumor response, since tumors with greater intratumoral heterogeneity are often assumed to have an aggressive biology [78]. However, no consensus exists as contradictory results have been published. These contradictory results may be explained by the lack of standardized methodology in extracting and analyzing radiomic features. Also the correlation between radiomic features and biological substrates has not been established definitively and could be different for the distinct tumor types.

The findings of this review demonstrate that the research field of radiomics is emerging, as 30% of included studies were published over the last year. These findings fit the trend of shifting from “one therapy fits all” to “personalized cancer treatment” in clinical practice. Moreover, rapid developments in advanced computed techniques have accelerated in the last decade. To our knowledge, this is the first review performed to provide an overview of radiomics in predicting tumor response in patients with GI cancer. Other reviews on radiomics have been published with regard to patients suffering from head and neck cancer, lung cancer, and breast cancer [13, 79–81]. The study of Granzier et al. focused on radiomics predicting tumor response in patients with breast cancer [79]. Similar to our review, entropy was the most frequently identified predictive feature reported and logistic regression the most frequently chosen model. In the study of Thawani et al., not only the potential of radiomics for diagnosis and prognosis was discussed but also the possibility to identify genetic mutations, which was called radiogenomics [80]. In accordance with the current findings, these reviews addressed the importance of standardized methodology as well. The great variation in methodology among studies is a barrier that needs to be resolved before radiomics can be implemented in clinical practice.

Radiomics is a promising method to tailor selection of patients for treatment strategies. If patients are likely to achieve complete pathological response after neoadjuvant treatment, a watch-and-wait strategy may be recommended instead of surgery. [3, 82, 83] Patients are likely to maintain a better quality of life with fewer treatment-related symptoms when often extensive surgery can be avoided, whereas exposure to unnecessary toxicity leading to adverse events may be spared in patients likely to have poor or no response to neoadjuvant treatment [6, 84]. Moreover, treatment strategy could be adjusted in a timely manner through early identification of patients who are likely to be unresponsive.

The potential utility of radiomics in clinical practice appears promising; however, most of the included studies lacked external validation, and results were often “over fit” due to small sample sizes. As a result, the predictive power may be overestimated. In addition, the majority of the studies lacked comparison of radiomics to standard of care (i.e., radiologists assessment), and only two studies performed a decision curve analysis [51, 62]. Therefore, the actual implementation of radiomics in clinical practice still seems a prospect for the future.

This review has some limitations. First, our findings are subject to publication bias. Only five studies were identified that described no significant association between radiomics and tumor response. This could either be a reflection of publication bias or a reflection of the truth. Second, even though the need for a meta-analysis of pooled metrics appeared evident, no meta-analysis stratified for tumor types could be performed, due to heterogeneity of included studies.

In future research, external validation of predictive models and cost-effectiveness analyses should be carried out, in order to define the role of radiomics in clinical practice. In addition, more standardized research in a prospectively manner is necessary to determine the added value of radiomics in predicting tumor response. Meta-analysis of data is needed to compare predictive radiomics models, but this is not feasible due to great variation in methodology. Therefore, we advocate external validation of current radiomics models, instead of conducting new models. The RQS and the Image Biomarkers Standardization Initiative (IBSI) are promising initiatives for standardization of radiomics research [10, 85].

In conclusion, radiomics is increasingly used to predict response to treatment in patients with GI cancer. Our review shows the great potential of this novel technique to help predict response to treatment and thereby to improve patient selection and early adjustment of treatment strategy in a non-invasive manner. However, as most studies lacked external validation, methodology varied considerably, and no models are implemented to predict response to treatment in patients suffering from GI cancer, yet in daily practice, future standardized research is warranted to determine the added value of radiomics in clinical practice.

## Supplementary Information

ESM 1 (DOCX 88 kb)

ESM 2 (DOCX 36 kb)

ESM 3 (DOCX 28 kb)

ESM 4 (DOCX 26 kb)

## Data Availability

Not applicable.
