# Supplementary material for: Advanced analytics and artificial intelligence in gastrointestinal cancer: a systematic review of radiomics predicting response to treatment
Source: Eur J Nucl Med Mol Imaging. 2020 Dec 16;48(6):1785–94. doi: 10.1007/s00259-020-05142-w (PMC8113210; doi:10.1007/s00259-020-05142-w)
Supplement: Supplementary file 1 — (DOCX 88 kb) [file 259_2020_5142_MOESM1_ESM.docx]

*European Journal of Nuclear Medicine and Molecular Imaging*

**Advanced analytics and artificial intelligence in gastrointestinal cancer: a systematic review of radiomics predicting response to treatment**

Nina J. Wesdorp^1*^; Tessa Hellingman^1*^; Elise P. Jansma^2^; Jan-Hein T. M. van Waesberghe^3^; Ronald Boellaard^4^; Cornelis J. A. Punt^5^; Joost Huiskens^6^; Geert Kazemier^1^

^1^Department of Surgery, Cancer Center Amsterdam, Amsterdam University Medical Centers, Vrije Universiteit, Amsterdam, The Netherlands; ^2^Department of Epidemiology and Biostatistics, Amsterdam University Medical Centers, Vrije Universiteit, Amsterdam, The Netherlands; ^3^Department of Radiology and Molecular Imaging, Cancer Center Amsterdam, Amsterdam University Medical Centers, Vrije Universiteit, Amsterdam, The Netherlands; ^4^Department of Radiology and Nuclear Medicine, Cancer Center Amsterdam, Amsterdam University Medical Centers, Vrije Universiteit, Amsterdam, The Netherlands; ^5^Julius Center for Health Sciences and Primary Care, University Medical Center Utrecht, Utrecht, The Netherlands; ^6^SAS Institute B.V., Huizen, The Netherlands; *^*^*shared first authorship. **Correspondence to:** Nina J. Wesdorp; Email: [n.wesdorp@amsterdamumc.nl](mailto:n.wesdorp@amsterdamumc.nl)

**Table 1. Study characteristics and predictive performance of radiomics**

|  | **Year** | **Treatment** | **Imaging** | | **Response** | **No. of** | **No. of parameters** | | **Best predictive performance** | | | | |
| --- | --- | --- | --- | --- | --- | --- | --- | --- | --- | --- | --- | --- | --- |
| **Reference** |  |  | **Modality** | **Timing** | **assessment** | **patients** | **Analysed** | **Selected** | **Model** | **Parameters** | **AUC** | **Accuracy** | **Other** |
| **OESOPHAGEAL CANCER** | | | | | | | | | | | | | |
| Beukinga | 2017 | CRT | PET/CT | pre | Pathologic | 97 | 88 | 3 | LASSO-LR | Histology | 0.76 | ·· | ·· |
| (19) |  |  |  |  |  |  |  |  |  | cT-stage |  |  |  |
|  |  |  |  |  |  |  |  |  |  | Run percentage (CT) |  |  |  |
| Beukinga | 2018 | CRT | PET/CT | pre + post | Pathologic | 73 | 346 | 2 | LASSO-LR | cT-stage | 0.81 | ·· | ·· |
| (18) |  |  |  |  |  |  |  |  |  | Post joint maximum |  |  |  |
| Chen | 2019 | CRT | PET/CT | pre | Pathologic | 42 | 23 | 1 | LR | Histogram Entropy | ·· | ·· | OR = 19.25 |
| (22) |  |  |  |  |  |  |  |  |  |  |  |  |  |
| Hou | 2017 | CRT | CT | pre | RECIST | 49 | 214 | 7 | ANN | Histogram2D Skewness | 0.80 | 0.92 | ·· |
| (27) |  |  |  |  |  |  |  |  |  | GLCM3D_Entropy |  |  |  |
|  |  |  |  |  |  |  |  |  |  | GLSZD 3D_Large zone emphasis |  |  |  |
|  |  |  |  |  |  |  |  |  |  | GLSZD 3D Small zone high |  |  |  |
|  |  |  |  |  |  |  |  |  |  | gray-level emphasis |  |  |  |
|  |  |  |  |  |  |  |  |  |  | Gabor mean square amplitude-42,-55 |  |  |  |
|  |  |  |  |  |  |  |  |  |  | Gabor mean square energy-26 |  |  |  |
| Hou | 2018 | CRT | MRI | pre | RECIST | 68 | 138 | 4 | ANN | GLCM_Inverse variance 0°/90° | ·· | 0.84 | ·· |
| (26) |  |  |  |  |  |  |  |  |  | GLCM Correlation 45° |  |  |  |
|  |  |  |  |  |  |  |  |  |  | Gabor_mean square energy-25 |  |  |  |
| Jin | 2019 | CRT | CT | ·· | RECIST | 94 | 63 | ·· | XGBoost | ·· | 0.69 | 0.71 | ·· |
| (20) |  |  |  |  |  |  |  |  | & PCA |  |  |  |  |
| Nakajo | 2017 | CRT | PET/CT | pre | Pathologic | 52 | 13 | ·· | ·· | Size-zone variability | 0.75 | 0.79 | ·· |
| (23) |  |  |  |  | + RECIST |  |  |  |  |  |  |  |  |
| Riyahi | 2018 | CRT | PET/CT | pre + post | Pathologic | 20 | 664 | 2 | SVM- | Median Jacobian | 0.94 | 0.94 | ·· |
| (29) |  |  |  |  |  |  |  |  | LASSO | Minimum Jacobian |  |  |  |
| Tixier | 2011 | CRT | PET/CT | pre | RECIST | 41 | 41 | ·· | ·· | Homogeneity | 0.89 | ·· | ·· |
| (24) |  |  |  |  |  |  |  |  |  |  |  |  |  |
| Yip | 2015 | CTx | CT | pre + post | Pathologic | 31 | 66 | ·· | ·· | Pre standard deviation (histogram) | ·· | ·· | ·· |
| (30) |  |  |  |  |  |  |  |  |  | Post standard deviation (histogram) |  |  |  |
| Yip | 2016 | CRT | PET/CT | pre + post | Pathologic | 54 | 8 | ·· | ·· | Delta Entropy | 0.79 | ·· | ·· |
| (25) |  |  |  |  |  |  |  |  |  |  |  |  |  |
| Ypsilantis | 2015 | CTx | PET | pre | Pathologic | 107 | 103 | ·· | 3S-CNN | ·· | ·· | 0.73 | ·· |
| (28) |  |  |  |  |  |  |  |  |  |  |  |  |  |
| Zhang | 2014 | CRT | PET/CT | pre + post | Pathologic | 20 | 169 | 17 | SVM | See ref (23) | 0.92 | ·· | ·· |
| (21) |  |  |  |  |  |  |  |  |  |  |  |  |  |
| **GASTROESOPHAGEAL CANCER** | | | | | | | | | | | | | |
| Giganti | 2017 | CTx | CT | pre | Pathologic | 34 | 107 | 3 | RF | Entropy (no filter) | 0·74 | 0·74 | OR = 60.94 |
| (36) |  |  |  |  |  |  |  |  |  |  |  |  |  |
| Klaassen | 2018 | CTx | CT | pre | Volumetric | 18 | 370 | ·· | RF | ·· | 0.79 | ·· | ·· |
| (31) |  |  |  |  |  |  |  |  |  |  |  |  |  |
| Wang | 2017 | CRT | CT | pre | Pathologic | 146 | 66 | 2 | LR | 3-point risk score | ·· | ·· | OR = 1.79 |
| (34) |  |  |  |  |  |  |  |  |  | (shape compactness and |  |  |  |
|  |  |  |  |  |  |  |  |  |  | pathologic grade differentiation) |  |  |  |
| **GASTRIC CANCER** | | | | | | | | | | | | | |
| Hou | 2018 | PLDRT | CT | pre | RECIST | 43 | 1117 | 4 | KNN | GLCM Cluster shade | ·· | 0.82 | ·· |
| (32) |  |  |  |  |  |  |  |  |  | LoG_2.5__GLSZM Large area low gray |  |  |  |
|  |  |  |  |  |  |  |  |  |  | level emphasis |  |  |  |
|  |  |  |  |  |  |  |  |  |  | W_LHL__GLSZM Size zone non |  |  |  |
|  |  |  |  |  |  |  |  |  |  | uniformity normalized |  |  |  |
|  |  |  |  |  |  |  |  |  |  | W_HHL__Skewness |  |  |  |
| Li | 2018 | CTx | CT | pre | Pathologic | 30 | 19985 | ·· | SBF-LDA | ·· | 0.72 | 0.79 | ·· |
| (35) |  |  |  |  |  |  |  |  | RF |  |  |  |  |
| **GASTROINTESTINAL STROMAL TUMORS** | | | | | | | | | | | | | |
| Ekert | 2019 | TKI | CT | longitudinal | Choi criteria | 25 | 92 | 4 | LR | GLCM Inverse difference | 0.83 | ·· | ·· |
| (33) |  |  |  |  |  |  |  |  |  | GLCM Inverse difference |  |  |  |
|  |  |  |  |  |  |  |  |  |  | normalized |  |  |  |
|  |  |  |  |  |  |  |  |  |  | NGTDM Coarseness |  |  |  |
|  |  |  |  |  |  |  |  |  |  | GLRLM Run length non-uniformity |  |  |  |
|  |  |  |  |  |  |  |  |  |  | normalized |  |  |  |
| **PRIMARY COLORECTAL CANCER** | | | | | | | | | | | | | |
| Aker | 2019 | CRT | MRI | post | Pathologic | 105 | 72 | ·· | ·· | Entropy | 0.88 | 0.88 | ·· |
| (37) |  |  |  |  |  |  |  |  |  |  |  |  |  |
| Bang | 2016 | CRT | PET | pre | Pathologic | 74 | 62 | 0 | LR | ·· | ·· | ·· | ·· |
| (38) |  |  |  |  |  |  |  |  |  |  |  |  |  |
| Bibault | 2018 | CRT | CT | pre | Pathologic | 95 | 1697 | 29 | DNN | ·· | 0.72 | 0.80 | ·· |
| (48) |  |  |  |  |  |  |  |  |  |  |  |  |  |
| Boldrini | 2019 | CRT | MRI | longitudinal | Clinical | 16 | 318 | 63 | ·· | Delta Grey level non-uniformity | ·· | ·· | ·· |
| (39) |  |  |  |  |  |  |  |  |  | Delta Least axis length |  |  |  |
| Bulens | 2019 | CRT | MRI | pre + post | Pathologic | 125 | 12790 | ·· | LASSO-LR | ·· | 0.86 | 0.82 | ·· |
| (49) |  |  |  |  |  |  |  |  |  |  |  |  |  |
| Caruso | 2018 | CRT | MRI | pre | Pathologic | 8 | 56 | 20 | LiR | See ref (42) | ·· | ·· | ·· |
| (40) |  |  |  |  |  |  |  |  |  |  |  |  |  |
| Chee | 2017 | CRT | CT | pre | Pathologic | 95 | 25 | 13 | ·· | Entropy (no filter, 1, 1.5) | ·· | ·· | ·· |
| (41) |  |  |  |  |  |  |  |  |  | Uniformity (no filter, 1, 1.5) |  |  |  |
|  |  |  |  |  |  |  |  |  |  | Kurtosis (filter 2.0) |  |  |  |
|  |  |  |  |  |  |  |  |  |  | Skewness (no filter, 2, 2.5) |  |  |  |
|  |  |  |  |  |  |  |  |  |  | SD (no filter, 1, 1.5) |  |  |  |
| Chidambaram | 2017 | CRT | MRI | pre | Pathologic | 78 | 8 | 1 | ·· | Tumor volume | ·· | ·· | ·· |
| (42) |  |  |  |  |  |  |  |  |  |  |  |  |  |
| Cusumano | 2018 | CRT | MRI | pre | Pathologic | 198 | ·· | 5 | LR | cT-stage | 0.79 | ·· | ·· |
| (50) |  |  |  |  |  |  |  |  |  | cN-stage |  |  |  |
|  |  |  |  |  |  |  |  |  |  | Skewness |  |  |  |
|  |  |  |  |  |  |  |  |  |  | Entropy |  |  |  |
|  |  |  |  |  |  |  |  |  |  | Max fractal dimension (40–100) |  |  |  |
| De Cecco | 2015 | CRT | MRI | pre + mid | Pathologic | 15 | 72 | ·· | ·· | Kurtosis | 0.91 | ·· | ·· |
| (44) |  |  |  |  |  |  |  |  |  |  |  |  |  |
| De Cecco | 2016 | CRT | CT | pre | Pathologic | 12 | 6 | 2 | ·· | Kurtosis | 0.86 | ·· | ·· |
| (43) |  |  |  |  |  |  |  |  |  | Ve | 0.86 | ·· | ·· |
| Ferrari | 2019 | CRT | MRI | longitudinal | Pathologic | 55 | 855 | 8 | RF | Min Entropy:post-CRT/pre-CRT (3D) | 0.86 | 0.79 | ·· |
| (51) |  |  |  |  |  |  |  |  |  | Min Entropy:post-CRT- pre-CRT (3D) |  |  |  |
|  |  |  |  |  |  |  |  |  |  | Min Entropy: post- CRT/mid-CRT (3D) |  |  |  |
|  |  |  |  |  |  |  |  |  |  | ROI Area Pre-CRT (2D) |  |  |  |
|  |  |  |  |  |  |  |  |  |  | Dissimilarity mid-CRT(2D) |  |  |  |
|  |  |  |  |  |  |  |  |  |  | Contrast mid-CRT (2D) |  |  |  |
|  |  |  |  |  |  |  |  |  |  | Homogeneity mid-CRT(2D) |  |  |  |
|  |  |  |  |  |  |  |  |  |  | Energy pre-CRT (2D) |  |  |  |
| Giannini | 2019 | CRT | MRI + | pre | Pathologic | 52 | 81 | 6 | LR | PET homogeneity | 0.86 | ·· | ·· |
| (52) |  |  | PET |  |  |  |  |  |  | PET contrast |  |  |  |
|  |  |  |  |  |  |  |  |  |  | PET 10th percentile |  |  |  |
|  |  |  |  |  |  |  |  |  |  | Glycolytic volume |  |  |  |
|  |  |  |  |  |  |  |  |  |  | Metabolic tumor volume |  |  |  |
|  |  |  |  |  |  |  |  |  |  | T2w MRI correlation 1 |  |  |  |
| Hamerla | 2019 | CRT | CT | pre | Pathologic | 169 | 1819 | 63 | RF | See ref (55) | ·· | 0.50 | ·· |
| (53) |  |  |  |  |  |  |  |  |  |  |  |  |  |
| Horvat | 2018 | CRT | MRI | post | Pathologic | 114 | 34 | 6 | RF | ·· | 0.93 | ·· | ·· |
| (54) |  |  |  |  |  |  |  |  |  |  |  |  |  |
| Hsu | 2016 | CRT | MRI or | pre | Pathologic | 122 | 10 | 3 | LR | cN stage | 0.80 | ·· | ·· |
| (45) |  |  | CT |  |  |  |  |  |  | Radiation dose |  |  |  |
|  |  |  |  |  |  |  |  |  |  | TCTV |  |  |  |
| Liu | 2017 | CRT | MRI | pre + post | Pathologic | 222 | 2269 | 31 | LR | ·· | 0.98 | 0.94 | ·· |
| (62) |  |  |  |  |  |  |  |  |  |  |  |  |  |
| Liu | 2019 | CRT | MRI | pre | Pathologic | 41 | 64 | 3 | LR | CorrelatD* | 0.97 | ·· | ·· |
| (63) |  |  |  |  |  |  |  |  |  | DifVarncADC |  |  |  |
|  |  |  |  |  |  |  |  |  |  | DifVarncD |  |  |  |
| Lovinfosse | 2018 | CRT | PET | pre | Pathologic | 66 | ·· | 2 | LR | Total lesion glycolysis | ·· | ·· | OR = 0.12 |
| (46) |  |  |  |  |  |  |  |  |  | RAS-mutational status | ·· | ·· | OR = 0.22 |
| Meng | 2018 | CRT | MRI | pre + mid | Pathologic | 59 | 14 | 3 | LR | Pre energy | 0.76 | ·· | ·· |
| (55) |  |  |  |  |  |  |  |  |  | Pre entropy |  |  |  |
|  |  |  |  |  |  |  |  |  |  | Pre uniformity |  |  |  |
| Nie | 2016 | CRT | MRI | pre | Pathologic | 48 | 103 | 2 | ANN | Mean ADC (DWI) | 0.84 | ·· | ·· |
| (56) |  |  |  |  |  |  |  |  |  | GLCM AutoCorrelation (DCE-MRI) |  |  |  |
| Shayesteh | 2019 | CRT | MRI | pre | Pathologic | 98 | 240 | 4 | Ensemble | ·· | 0.95 | 0.90 | ·· |
| (57) |  |  |  |  |  |  |  |  | (SVM, NN |  |  |  |  |
|  |  |  |  |  |  |  |  |  | BN, KNN) |  |  |  |  |
| Shi | 2019 | CRT | MRI | pre + mid | Pathologic | 51 | 204 | 5 | ANN | Mid DWI 50% | 0.86 | ·· | ·· |
| (58) |  |  |  |  |  |  |  |  |  | Mid ADC |  |  |  |
|  |  |  |  |  |  |  |  |  |  | Mid DWI contrast |  |  |  |
|  |  |  |  |  |  |  |  |  |  | Pre T2 homogeneity2 |  |  |  |
|  |  |  |  |  |  |  |  |  |  | Pre T1 contrast |  |  |  |
| Shu | 2019 | CRT | MRI | pre + mid | Pathologic | 136 | 12 | 3 | LR | Pre energy | 0.87 | ·· | ·· |
| (59) |  |  |  |  |  |  |  |  |  | Mid kurtosis |  |  |  |
|  |  |  |  |  |  |  |  |  |  | Mid entropy |  |  |  |
| V. | 2019 | CRT | MRI | pre | Pathologic | 133 | 2505 | 9 | LR | Dependence non uniformity | 0.77 | ·· | ·· |
| Griethuysen |  |  |  |  |  |  |  |  |  | normalized (ADC) |  |  |  |
| (60) |  |  |  |  |  |  |  |  |  | Gray level non uniformity (DWI) |  |  |  |
|  |  |  |  |  |  |  |  |  |  | Large area low gray level emphasis |  |  |  |
|  |  |  |  |  |  |  |  |  |  | (ADC) |  |  |  |
|  |  |  |  |  |  |  |  |  |  | Low gray level run emphasis (DWI) |  |  |  |
|  |  |  |  |  |  |  |  |  |  | Busyness (DWI) |  |  |  |
|  |  |  |  |  |  |  |  |  |  | Root mean squared (ADC) |  |  |  |
|  |  |  |  |  |  |  |  |  |  | Inverse difference normalized (ADC) |  |  |  |
|  |  |  |  |  |  |  |  |  |  | Inverse variance (DWI) |  |  |  |
|  |  |  |  |  |  |  |  |  |  | Root mean squared (DWI) |  |  |  |
| Yang | 2019 | CRT | MRI | post | Pathologic | 76 | 9 | 2 | LR | Mean ADC | 0.95 | ·· | ·· |
| (47) |  |  |  |  |  |  |  |  |  | Uniformity |  |  |  |
| Yi | 2019 | CRT | MRI | pre | Pathologic | 134 | 371 | 18 | SVM | ·· | 0.88 | 0.85 | ·· |
| (61) |  |  |  |  |  |  |  |  |  |  |  |  |  |
| **METASTATIC COLORECTAL CANCER** | | | | | | | | | | | | | |
| Ahn | 2016 | CTx | CT | pre | RECIST | 235 | 45 | 2 | LR | Skewness | 0.80 | ·· | OR = 6.739 |
| (64) |  |  |  |  |  |  |  |  |  |  |  |  |  |
| Beckers | 2018 | CTx | CT | pre | RECIST | 56 | 18 | 0 | ·· | ·· | ·· | ·· | ·· |
| (65) |  |  |  |  |  |  |  |  |  |  |  |  |  |
| Creasy | 2019 | HAI | CT | pre | Volumetric | 157 | 272 | 30 | LiR | ·· | ·· | ·· | MAPE = |
| (66) |  | (+ CTx) |  |  |  |  |  |  |  |  |  |  | 21.5% |
| Rao | 2016 | CTx | CT | pre + post | Pathologic | 21 | 36 | 0 | LR | ·· | ·· | ·· | ·· |
| (67) |  |  |  |  |  |  |  |  |  |  |  |  |  |
| V. Helden | 2018 | CTx | PET | pre | RECIST | 99 | 12 | ·· | LMEM | Mean entropy | 0.74 | ·· | ·· |
| (68) |  |  |  |  |  |  |  |  |  |  |  |  |  |
| Zhang | 2018 | CTx | MRI | pre | Diameter | 26 | 10 | 2 | LR | Variance | 0.81 | ·· | ·· |
| (69) |  |  |  |  | change |  |  |  |  | Angular second moment |  |  |  |
| **HEPATOCELLULAR CARCINOMA** | | | | | | | | | | | | | |
| Cozzi | 2017 | VMAT | CT | pre | RECIST | 106 | 35 | 1 | LR | Energy | 0.67 | ·· | ·· |
| (70) |  |  |  |  |  |  |  |  |  |  |  |  |  |
| Kloth | 2017 | TACE | CT | pre + post | mRECIST | 28 | 108 | 1 | ·· | Uniformity skewness | 0.80 | ·· | ·· |
| (71) |  |  |  |  |  |  |  |  |  |  |  |  |  |
| Park | 2017 | TACE | CT | pre | mRECIST | 96 | 44 | 10 | LR | Homogeneity | 0.72 | ·· | ·· |
| (72) |  |  |  |  |  |  |  |  |  |  |  |  |  |
| Yu | 2018 | TACE/ | MRI | pre + post | RECICL | 89 | 20 | 6 | LR | Post skewness | 0.76 | ·· | ·· |
| (73) |  | HIFU |  |  |  |  |  |  |  |  |  |  |  |
| **PANCREATIC CANCER** | | | | | | | | | | | | | |
| Borhani | 2019 | CTx | CT | pre + post | Pathologic | 39 | 108 | 2 | LR | Chemotherapy regimen | ·· | ·· | OR = 13.5 |
| (74) |  |  |  |  |  |  |  |  |  | Mean positive pixel |  |  | OR = 1.06 |
| Ciaravino | 2018 | CTx/ | CT | pre + post | Downstaged | 31 | 10 | ·· | ·· | Kurtosis | ·· | ·· | ·· |
| (75) |  | CRT |  |  |  |  |  |  |  |  |  |  |  |
| Nasief | 2019 | CRT | CT | longitudinal | Pathologic | 90 | 1300 | 3 | BNN | Kurtosis | 0.94 | 0.90 | ·· |
| (76) |  |  |  |  |  |  |  |  |  | Coarseness |  |  |  |
|  |  |  |  |  |  |  |  |  |  | NESTD |  |  |  |
| Yoo | 2019 | CTx | PET/CT | pre + post | RECIST | 63 | 54 | ·· | ·· | Metabolic tumor volume | ·· | ·· | ·· |
| (77) |  |  |  |  |  |  |  |  |  |  |  |  |  |

·· = not reported

**Abbreviations:** ADC = apparent diffusion coefficient, ANN = artificial neural network, AUC = area under the receiver operator characteristic curve, BNN = Bayesian neural network, CNN = convolutional neural network, CRT = chemoradiotherapy, CT = computed tomography, cT-stage = clinical tumor stage, cN-stage = clinical nodal stage, CTx = chemotherapy, DCE = dynamic contrast enhanced, DNN = deep neural network, DWI = diffusion weighted imaging, GB = gradient boosting classifier, GLCM = Gray-level co-occurrence matrix, GLRLM = Gray-level run-length matrix, GLSZM = Gray-level size zone matrix, HAI = hepatic artery infusion, HIFU = high-intensity focused ultrasound ablation, KNN = k-nearest neighbor, LASSO-LR = least absolute shrinkage selection operator and logistic regression, LiR = linear regression model, LMEM = linear mixed effect models, LoG = laplacian of gaussian filter, LR = logistic regression model, MAPE = mean absolute prediction error, mRECIST = modified Response Evaluation Criteria in Solid Tumors, MRI = magnetic resonance imaging, NESTD = normalized entropy to standard deviation difference, NGTDM = Neighborhood gray-tone difference matrix, NN = neural network, OR = odds ratio, PCA = principal component analysis, PET = positron emission tomography, PLDRT = Pulsed low dose rate radiotherapy, RECIST = Response Evaluation Criteria in Solid Tumors, RECICL = Response Evaluation in Cancer of the Liver, RF = random forest classifier, ROI = region of interest, SBF-LDA = selection by filter based linear discriminant analysis, SD = standard deviation, STD = stable disease, SUV = standardized uptake values, SVM = supporter vector machine, SVM-LASSO = support vector machine classifier coupled with a least absolute shrinkage and selection operator, s, TACE = transarterial chemoembolization, TCTV = tumor compactness-corrected tumor volume, TKI = tyrosine kinase inhibitors, VMAT = volumetric modulated arc therapy, W = wavelet transformation, XGBoost = extreme gradient boosting algorithm, 2D = two-dimensional, 3D = three-dimensional.
