# Supplementary material for: Advanced analytics and artificial intelligence in gastrointestinal cancer: a systematic review of radiomics predicting response to treatment
Source: Eur J Nucl Med Mol Imaging. 2020 Dec 16;48(6):1785–94. doi: 10.1007/s00259-020-05142-w (PMC8113210; doi:10.1007/s00259-020-05142-w)
Supplement: Supplementary file 2 — (DOCX 36 kb) [file 259_2020_5142_MOESM2_ESM.docx]

*European Journal of Nuclear Medicine and Molecular Imaging*

**Advanced analytics and artificial intelligence in gastrointestinal cancer: a systematic review of radiomics predicting response to treatment**

Nina J. Wesdorp^1*^; Tessa Hellingman^1*^; Elise P. Jansma^2^; Jan-Hein T. M. van Waesberghe^3^; Ronald Boellaard^4^; Cornelis J. A. Punt^5^; Joost Huiskens^6^; Geert Kazemier^1^

*^*^*shared first authorship ^1^Department of Surgery, Cancer Center Amsterdam, Amsterdam University Medical Centers, Vrije Universiteit, Amsterdam, The Netherlands; ^2^Department of Epidemiology and Biostatistics, Amsterdam University Medical Centers, Vrije Universiteit, Amsterdam, The Netherlands; ^3^Department of Radiology and Molecular Imaging, Cancer Center Amsterdam, Amsterdam University Medical Centers, Vrije Universiteit, Amsterdam, The Netherlands; ^4^Department of Radiology and Nuclear Medicine, Cancer Center Amsterdam, Amsterdam University Medical Centers, Vrije Universiteit, Amsterdam, The Netherlands; ^5^Julius Center for Health Sciences and Primary Care, University Medical Center Utrecht, Utrecht, The Netherlands; ^6^SAS Institute B.V., Huizen, The Netherlands. **Correspondence to:** Nina J. Wesdorp; Email: [n.wesdorp@amsterdamumc.nl](mailto:n.wesdorp@amsterdamumc.nl)

**Table 2. Radiomics quality score of included studies**

| **Study (ref*)*** | Image protocol | Multiple segmentations | Inter-scanner differences | Imaging multiple times | Feature reduction^a^ | Non-radiomic features^b^ | Biological correlates | Cut-offs^c^ | Discriminative statistics^d^ | Calibration^e^ | Prospective | Validation | Compare 'gold standard' | Clinical utility | Cost-effectiveness | Open science^f^ | **Total points (%)** |
| --- | --- | --- | --- | --- | --- | --- | --- | --- | --- | --- | --- | --- | --- | --- | --- | --- | --- |
| **OESOPHAGEAL CANCER** | | | | | | | | | | | | | | | | | |
| Beukinga ^(19)^ | 2 | 1 | 0 | 0 | 3 | 1 | 1 | 0 | 2 | 2 | 0 | -5 | 2 | 2 | 0 | 0 | 11 (31%) |
| Beukinga ^(18)^ | 2 | 1 | 0 | 0 | 3 | 1 | 1 | 1 | 2 | 2 | 0 | -5 | 2 | 2 | 0 | 0 | 12 (33%) |
| Chen ^(22)^ | 1 | 0 | 0 | 0 | -3 | 1 | 1 | 0 | 1 | 0 | 0 | 2 | 2 | 2 | 0 | 0 | 7 (19%) |
| Hou ^(27)^ | 1 | 1 | 0 | 0 | 3 | 0 | 0 | 0 | 2 | 2 | 0 | 2 | 0 | 2 | 0 | 0 | 13 (36%) |
| Hou ^(26)^ | 1 | 1 | 0 | 0 | 3 | 0 | 0 | 0 | 1 | 2 | 0 | 2 | 0 | 2 | 0 | 1^(II)^ | 13 (36%) |
| Jin ^(20)^ | 1 | 1 | 0 | 0 | 3 | 1 | 0 | 1 | 2 | 2 | 0 | 2 | 0 | 2 | 0 | 1^(II)^ | 16 (44%) |
| Nakajo ^(23)^ | 1 | 0 | 0 | 0 | -3 | 1 | 1 | 0 | 1 | 0 | 0 | -5 | 2 | 2 | 0 | 0 | 0 |
| Riyahi ^(29)^ | 0 | 0 | 0 | 0 | 3 | 0 | 1 | 0 | 2 | 0 | 0 | -5 | 0 | 2 | 0 | 0 | 3 (8%) |
| Tixier ^(24)^ | 1 | 0 | 0 | 0 | -3 | 0 | 0 | 0 | 1 | 0 | 0 | -5 | 0 | 2 | 0 | 0 | -4 (-11%) |
| Yip ^(30)^ | 1 | 1 | 0 | 0 | 3 | 0 | 1 | 0 | 0 | 0 | 0 | -5 | 0 | 2 | 0 | 0 | 3 (8%) |
| Yip ^(25)^ | 1 | 0 | 0 | 0 | 3 | 0 | 1 | 1 | 1 | 0 | 0 | -5 | 0 | 2 | 0 | 0 | 4 (11%) |
| Ypsilantis ^(28)^ | 0 | 0 | 0 | 0 | 3 | 0 | 1 | 0 | 0 | 2 | 0 | -5 | 0 | 2 | 0 | 0 | 3 (8%) |
| Zhang ^(21)^ | 0 | 0 | 0 | 0 | 3 | 1 | 1 | 0 | 2 | 0 | 0 | -5 | 0 | 2 | 0 | 0 | 4 (11%) |
| **GASTRO-OESOPHAGEAL CANCER** | | | | | | | | | | | | | | | | | |
| Giganti ^(36)^ | 1 | 1 | 0 | 0 | 3 | 0 | 1 | 0 | 2 | 2 | 0 | -5 | 0 | 2 | 0 | 0 | 7 (19%) |
| Klaassen ^(31)^ | 1 | 0 | 0 | 0 | 3 | 0 | 0 | 0 | 2 | 0 | 0 | -5 | 0 | 2 | 0 | 0 | 3 (8%) |
| Wang ^(34)^ | 1 | 0 | 0 | 0 | 3 | 1 | 1 | 0 | 1 | 0 | 0 | 2 | 0 | 2 | 0 | 0 | 11 (31%) |
| **GASTRIC CANCER** | | | | | | | | | | | | | | | | | |
| Hou ^(32)^ | 1 | 1 | 0 | 0 | 3 | 0 | 0 | 0 | 1 | 2 | 0 | 2 | 0 | 2 | 0 | 1^(II)^ | 13 (36%) |
| Li ^(35)^ | 1 | 0 | 0 | 0 | 3 | 0 | 1 | 0 | 2 | 1 | 0 | -5 | 0 | 2 | 0 | 0 | 5 (14%) |
| **GASTROINTESTINAL STROMAL TUMORS** | | | | | | | | | | | | | | | | | |
| Ekert ^(33)^ | 1 | 0 | 0 | 0 | 3 | 1 | 1 | 0 | 1 | 0 | 0 | -5 | 0 | 2 | 0 | 0 | 4 (11%) |
| **PRIMARY COLORECTAL CANCER** | | | | | | | | | | | | | | | | | |
| Aker ^(37)^ | 1 | 1 | 0 | 0 | -3 | 0 | 1 | 0 | 1 | 0 | 0 | -5 | 2 | 2 | 0 | 0 | 0 |
| Bang ^(38)^ | 1 | 0 | 0 | 0 | 3 | 1 | 1 | 1 | 0 | 0 | 0 | -5 | 0 | 2 | 0 | 0 | 4 (11%) |
| Bibault ^(48)^ | 1 | 1 | 0 | 0 | 3 | 1 | 1 | 1 | 2 | 2 | 0 | -5 | 2 | 2 | 0 | 3^(IIIV)^ | 14 (39%) |
| Boldrini ^(39)^ | 1 | 1 | 0 | 0 | -3 | 0 | 0 | 1 | 0 | 0 | 0 | -5 | 0 | 2 | 0 | 0 | -3 (-8%) |
| Bulens ^(49)^ | 1 | 1 | 0 | 0 | 3 | 0 | 1 | 1 | 2 | 2 | 7 | 2 | 0 | 2 | 0 | 1^(I)^ | 23 (64%) |
| Caruso ^(40)^ | 1 | 1 | 0 | 0 | -3 | 0 | 0 | 1 | 0 | 0 | 0 | -5 | 0 | 2 | 0 | 1^(II)^ | -2 (-6%) |
| Chee ^(41)^ | 1 | 1 | 0 | 0 | -3 | 1 | 1 | 0 | 0 | 0 | 0 | -5 | 0 | 2 | 0 | 0 | -2 (-6%) |
| Chidambaram ^(42)^ | 1 | 0 | 0 | 0 | -3 | 0 | 1 | 1 | 0 | 0 | 0 | -5 | 0 | 2 | 0 | 0 | -3 (-8%) |
| Cusumano ^(50)^ | 1 | 1 | 0 | 0 | 3 | 1 | 1 | 1 | 1 | 0 | 0 | 3 | 0 | 2 | 0 | 0 | 14 (39%) |
| De Cecco ^(44)^ | 1 | 0 | 0 | 0 | -3 | 0 | 1 | 0 | 1 | 0 | 7 | -5 | 0 | 2 | 0 | 0 | 4 (11%) |
| De Cecco ^(43)^ | 1 | 0 | 0 | 0 | -3 | 0 | 1 | 0 | 1 | 0 | 7 | -5 | 0 | 2 | 0 | 0 | 4 (11%) |
| Ferrari ^(51)^ | 1 | 0 | 0 | 0 | 3 | 0 | 1 | 1 | 2 | 0 | 0 | -5 | 2 | 2 | 0 | 1^(II)^ | 8 (22%) |
| Giannini ^(52)^ | 1 | 0 | 0 | 0 | 3 | 0 | 1 | 0 | 2 | 0 | 0 | -5 | 0 | 2 | 0 | 0 | 4 (11%) |
| Hamerla ^(53)^ | 1 | 1 | 0 | 0 | 3 | 0 | 1 | 1 | 2 | 2 | 0 | -5 | 0 | 2 | 0 | 0 | 8 (22%) |
| Horvat ^(54)^ | 1 | 0 | 0 | 0 | 3 | 0 | 1 | 1 | 2 | 0 | 0 | -5 | 2 | 2 | 0 | 1^(II)^ | 8 (22%) |
| Hsu ^(45)^ | 1 | 1 | 0 | 0 | 3 | 1 | 1 | 0 | 1 | 0 | 0 | -5 | 0 | 2 | 0 | 0 | 5 (14%) |
| Liu ^(62)^ | 1 | 1 | 0 | 0 | 3 | 1 | 1 | 1 | 2 | 2 | 0 | 2 | 0 | 2 | 0 | 1^(I)^ | 17 (47%) |
| Liu ^(63)^ | 1 | 0 | 0 | 0 | 3 | 0 | 1 | 0 | 1 | 0 | 7 | -5 | 0 | 2 | 0 | 0 | 10 (28%) |
| Lovinfosse ^(46)^ | 2 | 0 | 0 | 0 | 3 | 1 | 1 | 0 | 2 | 0 | 0 | -5 | 0 | 2 | 0 | 1^(I)^ | 7 (19%) |
| Meng ^(55)^ | 1 | 1 | 0 | 0 | 3 | 0 | 1 | 0 | 1 | 0 | 0 | -5 | 0 | 2 | 0 | 0 | 4 (11%) |
| Nie ^(56)^ | 1 | 0 | 0 | 0 | 3 | 0 | 1 | 1 | 2 | 0 | 0 | -5 | 0 | 2 | 0 | 0 | 5 (14%) |
| Shayesteh ^(57)^ | 1 | 1 | 0 | 0 | 3 | 0 | 1 | 1 | 1 | 1 | 7 | 2 | 0 | 2 | 0 | 1^(II)^ | 21 (58%) |
| Shi ^(58)^ | 1 | 1 | 0 | 0 | 3 | 0 | 1 | 1 | 2 | 2 | 0 | -5 | 2 | 2 | 0 | 0 | 10 (28%) |
| Shu ^(59)^ | 1 | 1 | 0 | 0 | 3 | 0 | 1 | 0 | 1 | 0 | 7 | -5 | 0 | 2 | 0 | 0 | 11 (31%) |
| V.  Griethuysen ^(60)^ | 1 | 1 | 1 | 0 | 3 | 0 | 1 | 1 | 2 | 0 | 0 | 3 | 2 | 2 | 0 | 0 | 17 (47%) |
| Yang ^(47)^ | 1 | 1 | 0 | 0 | 3 | 0 | 1 | 0 | 1 | 0 | 0 | -5 | 0 | 2 | 0 | 0 | 4 (11%) |
| Yi ^(61)^ | 1 | 1 | 0 | 0 | 3 | 1 | 1 | 1 | 1 | 1 | 0 | 2 | 0 | 2 | 0 | 0 | 14 (39%) |
| **METASTATIC COLORECTAL CANCER** | | | | | | | | | | | | | | | | | |
| Ahn ^(64)^ | 1 | 1 | 1 | 0 | 3 | 0 | 0 | 1 | 1 | 0 | 0 | 2 | 0 | 2 | 0 | 0 | 12 (33%) |
| Beckers ^(65)^ | 1 | 0 | 0 | 0 | 3 | 1 | 0 | 1 | 0 | 0 | 0 | -5 | 0 | 2 | 0 | 1^(II)^ | 4 (11%) |
| Creasy ^(66)^ | 1 | 0 | 0 | 0 | 3 | 1 | 0 | 1 | 0 | 1 | 0 | 2 | 0 | 2 | 0 | 0 | 11 (31%) |
| Rao ^(67)^ | 1 | 0 | 0 | 0 | -3 | 1 | 1 | 1 | 0 | 0 | 0 | -5 | 2 | 2 | 0 | 0 | 0 |
| V. Helden ^(68)^ | 2 | 0 | 0 | 0 | 3 | 1 | 0 | 1 | 1 | 0 | 0 | -5 | 2 | 2 | 0 | 0 | 7 (19%) |
| Zhang ^(69)^ | 1 | 1 | 0 | 0 | 3 | 0 | 0 | 0 | 1 | 0 | 0 | -5 | 0 | 2 | 0 | 0 | 3 (8%) |
| **HEPATOCELLULAR CARCINOMA** | | | | | | | | | | | | | | | | | |
| Cozzi ^(70)^ | 1 | 0 | 0 | 0 | 3 | 1 | 0 | 0 | 1 | 1 | 0 | -5 | 0 | 2 | 0 | 0 | 4 (11%) |
| Kloth ^(71)^ | 1 | 1 | 0 | 0 | 3 | 0 | 0 | 0 | 1 | 0 | 0 | -5 | 0 | 2 | 0 | 0 | 3 (8%) |
| Park ^(72)^ | 1 | 1 | 0 | 0 | 3 | 0 | 0 | 0 | 1 | 0 | 0 | -5 | 0 | 2 | 0 | 0 | 3 (8%) |
| Yu ^(73)^ | 1 | 0 | 0 | 0 | -3 | 0 | 0 | 0 | 1 | 0 | 0 | -5 | 0 | 2 | 0 | 0 | -4 (-11%) |
| **PANCREATIC CANCER** | | | | | | | | | | | | | | | | | |
| Borhani ^(74)^ | 1 | 0 | 0 | 0 | -3 | 1 | 1 | 1 | 0 | 0 | 0 | -5 | 0 | 2 | 0 | 0 | -2 (-6%) |
| Ciaravino ^(75)^ | 1 | 0 | 0 | 0 | -3 | 0 | 0 | 1 | 0 | 0 | 0 | -5 | 0 | 2 | 0 | 0 | -4 (-11%) |
| Nasief ^(76)^ | 1 | 1 | 1 | 1 | 3 | 0 | 1 | 1 | 2 | 2 | 0 | 2 | 0 | 2 | 0 | 2^(I,IV)^ | 19 (53%) |
| Yoo ^(77)^ | 1 | 0 | 0 | 0 | 3 | 1 | 0 | 0 | 1 | 0 | 7 | -5 | 0 | 2 | 0 | 1^(II)^ | 11 (31%) |

^a^ Feature reduction or adjustment for multiple testing
^b^ Multivariable analysis with non radiomics features
^c^ Cut-off analyses with risk groups determined by either the median, a previously published or report of a continuous risk variable
^d^ Discriminative statistics and resampling method applied
^e^ Calibration statistics and resampling method applied
^f^ Open science and data with open source code^(I)^, segmentation or feature extraction software^(II)^, scans^(III)^, and calculated features with representative ROIs^(IV)^.
