# Supplementary material for: Advanced analytics and artificial intelligence in gastrointestinal cancer: a systematic review of radiomics predicting response to treatment
Source: Eur J Nucl Med Mol Imaging. 2020 Dec 16;48(6):1785–94. doi: 10.1007/s00259-020-05142-w (PMC8113210; doi:10.1007/s00259-020-05142-w)
Supplement: Supplementary file 3 — (DOCX 28 kb) [file 259_2020_5142_MOESM3_ESM.docx]

*European Journal of Nuclear Medicine and Molecular Imaging*

**Advanced analytics and artificial intelligence in gastrointestinal cancer: a systematic review of radiomics predicting response to treatment**

Nina J. Wesdorp^1*^; Tessa Hellingman^1*^; Elise P. Jansma^2^; Jan-Hein T. M. van Waesberghe^3^; Ronald Boellaard^4^; Cornelis J. A. Punt^5^; Joost Huiskens^6^; Geert Kazemier^1^

^1^Department of Surgery, Cancer Center Amsterdam, Amsterdam University Medical Centers, Vrije Universiteit, Amsterdam, The Netherlands; ^2^Department of Epidemiology and Biostatistics, Amsterdam University Medical Centers, Vrije Universiteit, Amsterdam, The Netherlands; ^3^Department of Radiology and Molecular Imaging, Cancer Center Amsterdam, Amsterdam University Medical Centers, Vrije Universiteit, Amsterdam, The Netherlands; ^4^Department of Radiology and Nuclear Medicine, Cancer Center Amsterdam, Amsterdam University Medical Centers, Vrije Universiteit, Amsterdam, The Netherlands; ^5^Julius Center for Health Sciences and Primary Care, University Medical Center Utrecht, Utrecht, The Netherlands; ^6^SAS Institute B.V., Huizen, The Netherlands; *^*^*shared first authorship. **Correspondence to:** Nina J. Wesdorp; Email: [n.wesdorp@amsterdamumc.nl](mailto:n.wesdorp@amsterdamumc.nl)

**Search strategy Pubmed, Embase, and Cochrane**

PubMed

| **Search** | **PubMed Query 12-12-2019** | **Items found** |
| --- | --- | --- |
| [#5](https://www.ncbi.nlm.nih.gov/pubmed) | Search **#4 NOT (animals[mh] NOT humans[mh])** | [773](https://www.ncbi.nlm.nih.gov/pubmed/?cmd=HistorySearch&querykey=5) |
| [#4](https://www.ncbi.nlm.nih.gov/pubmed) | Search **#1 AND #2 AND #3** | [789](https://www.ncbi.nlm.nih.gov/pubmed/?cmd=HistorySearch&querykey=4) |
| [#3](https://www.ncbi.nlm.nih.gov/pubmed) | Search **"Outcome Assessment, Health Care"[Mesh] OR "Outcome Assessment"[tiab] OR treatment outcome[tiab] OR Tumor respons*[tiab] OR tumour respons*[tiab] OR chemo respons*[tiab] OR treatment respons*[tiab] OR therapy respons*[tiab] OR chemotherapy respons*[tiab] OR response assess*[tiab] OR response evaluat*[tiab] OR response prediction[tiab] OR predictive respons*[tiab] OR predicting respons*[tiab] OR outcome prediction*[tiab] OR predictive outcome*[tiab] OR predicting outcome*[tiab]** | [1085796](https://www.ncbi.nlm.nih.gov/pubmed/?cmd=HistorySearch&querykey=3) |
| [#2](https://www.ncbi.nlm.nih.gov/pubmed) | Search **"Artificial Intelligence"[Mesh] OR Computational Intelligen*[tiab] OR Machine Intelligen*[tiab] OR Computer Reasoning[tiab] OR Artificial Intelligen*[tiab] OR Computer Vision[tiab] OR Machine learning[tiab] OR fuzzy logic[tiab] OR "Decision Making, Computer-Assisted"[Mesh:NoExp] OR "Diagnosis, Computer-Assisted"[Mesh] OR Computer-Assisted Image Interpretation*[tiab] OR Computer-Assisted Radiographic Image Interpretation*[tiab] OR computer assisted diagnos*[tiab] OR computer-assisted decision making[tiab] OR automatic diagnos*[tiab] OR computer diagnos*[tiab] OR computer-assisted diagnos*[tiab] OR radiomic*[tiab] OR radiogenomic*[tiab] OR "Data Mining"[Mesh] OR "Data Mining"[tiab] OR "Datamining"[tiab] OR "Big Data"[Mesh] OR "Big Data"[tiab] OR "bigdata"[tiab] OR texture anal*[tiab] OR textural anal*[tiab] OR "Data Science"[Mesh] OR Data Science*[tiab] OR Data Analytic*[tiab] OR Data-Driven Science*[tiab] OR "Big Data"[Mesh] OR "Datasets as Topic"[Mesh] OR feature extracti*[tiab] OR quantitative image analy*[tiab] OR quantitative image feature*[tiab]** | [194008](https://www.ncbi.nlm.nih.gov/pubmed/?cmd=HistorySearch&querykey=2) |
| [#1](https://www.ncbi.nlm.nih.gov/pubmed) | Search **"Digestive System Neoplasms"[Mesh] OR Digestive System Neoplas*[tiab] OR Gastrointestinal Neoplas*[tiab] OR gastrointestinal carcinoma*[tiab] OR Gastrointestinal Tract Cancer*[tiab] OR Gastrointestinal Cancer*[tiab] OR "Esophageal Neoplasms"[Mesh] OR Esophageal Neoplasm*[tiab] OR Esophageal cancer*[tiab] OR Oesophageal Neoplasm*[tiab] OR Oesophageal cancer*[tiab] OR Esophageal carcinoma*[tiab] OR Esophageal tumor*[tiab] OR Esophageal tumour*[tiab] OR Oesophageal carcinoma*[tiab] OR Oesophageal tumor*[tiab] OR Oesophageal tumour*[tiab] OR "Stomach Neoplasms"[Mesh] OR Stomach Neoplasm*[tiab] OR Gastric Neoplasm*[tiab] OR "Cancer of Stomach"[tiab] OR Stomach Cancer*[tiab] OR Gastric Cancer*[tiab] OR "Cancer of the Stomach"[tiab] OR gastric carcinoma*[tiab] OR gastric tumor*[tiab] OR gastric tumour*[tiab] OR Intestinal Neoplasm*[tiab] OR Intestines Neoplasm*[tiab] OR Cancer of Intestine*[tiab] OR Intestines Cancer*[tiab] OR Cancer of the Intestine*[tiab] OR Intestinal Cancer*[tiab] OR small bowel cancer*[tiab] OR Duodenal Neoplasm*[tiab] OR duodenum neoplasm*[tiab] OR Duodenal Cancer*[tiab] OR Cancer of the Duodenum[tiab] OR Cancer of Duodenum[tiab] OR Duodenum Cancer*[tiab] OR duodenum carcinoma*[tiab] OR duodenal carcinoma*[tiab] OR duodenum tumor*[tiab] OR duodenal tumor*[tiab] OR duodenum tumour*[tiab] OR duodenal tumour*[tiab] OR "duodenal metastasis"[tiab] OR jejunal cancer*[tiab] OR jejunal neoplasm*[tiab] OR jejunal carcinoma*[tiab] OR jejunal tumor*[tiab] OR jejunal tumour*[tiab] OR jejunal metastas*[tiab] OR ileal cancer*[tiab] OR ileal neoplasm*[tiab] OR ileal carcinoma*[tiab] OR ileal tumor*[tiab] OR ileal tumour*[tiab] OR ileal metastas*[tiab] OR ileocecal cancer*[tiab] OR ileocecal carcinoma*[tiab] OR ileocecal tumor*[tiab] OR cecal cancer*[tiab] OR cecal neoplasm*[tiab] OR cecal carcinoma*[tiab] OR cecal tumor*[tiab] OR peritoneal metastas*[tiab] OR "Colonic Neoplasms"[Mesh] OR Colonic Neoplas*[tiab] OR Colon Neoplas*[tiab] OR "Cancer of Colon"[tiab] OR "Cancer of the Colon"[tiab] OR Colon Cancer*[tiab] OR Colonic Cancer*[tiab] OR Colon tumour*[tiab] OR colon tumor*[tiab] OR colonic tumour*[tiab] OR colonic tumor*[tiab] OR "Colorectal Neoplasms"[Mesh] OR Colorectal Neoplas*[tiab] OR Colorectal Tumor*[tiab] OR Colorectal Tumour*[tiab] OR Colorectal Carcinoma*[tiab] OR Colorectal Cancer*[tiab] OR colorectal metastas*[tiab] OR colorectal lung metastas*[tiab] OR metastatic colorectal*[tiab] OR rectal cancer*[tiab] OR rectal neoplasm*[tiab] OR rectal tumor*[tiab] OR rectal tumour*[tiab] OR rectal metastas*[tiab] OR rectal carcinoma*[tiab] OR "Pancreatic Neoplasms"[Mesh:NoExp] OR "Carcinoma, Pancreatic Ductal"[Mesh] OR Pancreatic Neoplasm*[tiab] OR Pancreas Neoplasm*[tiab] OR "Cancer of Pancreas"[tiab] OR Pancreas Cancer*[tiab] OR Pancreatic Cancer*[tiab] OR "Cancer of the Pancreas"[tiab] OR pancreas tumor*[tiab] OR pancreas tumour*[tiab] OR pancreas metastas*[tiab] OR pancreas carcinoma*[tiab] OR pancreatic tumor*[tiab] OR pancreatic tumour*[tiab] OR pancreatic metastas*[tiab] OR pancreatic carcinoma*[tiab] OR "Liver Neoplasms"[Mesh] OR Liver Neoplas*[tiab] OR Hepatic Neoplasm*[tiab] OR Cancer of Liver[tiab] OR "Cancer of the Liver"[tiab] OR Hepatic Cancer*[tiab] OR Liver Cancer*[tiab] OR Liver Cell Adenoma*[tiab] OR liver cell carcinoma*[tiab] OR liver carcinoma*[tiab] OR liver cell cancer*[tiab] OR liver cell tumor*[tiab] OR liver cell tumour*[tiab] OR liver metastas*[tiab] OR hepatic metastas*[tiab] OR hepatic colorectal metastas*[tiab] OR colorectal liver metastas*[tiab] OR liver tumor*[tiab] OR liver tumour*[tiab] OR Biliary Tract Neoplas*[tiab] OR gallbladder neoplas*[tiab] OR gallbladder cancer*[tiab] OR gallbladder metastas*[tiab] OR gallbladder tumor*[tiab] OR gallbladder tumour*[tiab] OR gallbladder carcinoma*[tiab] OR bile duct neoplas*[tiab] OR bile duct cancer*[tiab] OR "Cancer of the Bile Duct"[tiab] OR "Cancer of Bile Duct"[tiab] OR bile duct carcinoma*[tiab] OR "Gastrointestinal Stromal Tumors"[Mesh] OR Gastrointestinal Stromal Tumor*[tiab] OR Gastrointestinal Stromal Tumour*[tiab] OR Gastrointestinal Stromal Neoplasm*[tiab] OR Gastrointestinal Stromal Cancer*[tiab] OR Gastrointestinal stromal sarcoma*[tiab] OR Gastrointestinal neuroendocrine neoplasm*[tiab] OR Gastrointestinal neuroendocrine tumor*[tiab] OR Gastrointestinal neuroendocrine tumour*[tiab] OR Gastrointestinal neuroendocrine carcinoma*[tiab] OR pancreatic neuroendocrine tumor*[tiab] OR pancreatic neuroendocrine tumour*[tiab] OR pancreatic neuroendocrine carcinoma*[tiab] OR pancreatic neuroendocrine neoplasm*[tiab] OR gastric neuroendocrine tumor*[tiab] OR gastric neuroendocrine tumour*[tiab] OR gastric neuroendocrine carcinoma*[tiab] OR gastric neuroendocrine neoplasm*[tiab] OR intestinal neuroendocrine tumor*[tiab] OR intestinal neuroendocrine tumor*[tiab] OR intestinal neuroendocrine neoplasm*[tiab] OR esophageal neuroendocrine tumor*[tiab] OR esophageal neuroendocrine carcinoma*[tiab] OR esophageal neuroendocrine neoplasm*[tiab] OR rectal neuroendocrine tumor*[tiab] OR rectal neuroendocrine tumour*[tiab] OR rectal neuroendocrine carcinoma*[tiab] OR rectal neuroendocrine neoplasm*[tiab] OR hepatic neuroendocrine tumor*[tiab] OR hepatic neuroendocrine tumour*[tiab] OR hepatic neuroendocrine carcinoma*[tiab] OR hepatic neuroendocrine neoplasm*[tiab]** | [702460](https://www.ncbi.nlm.nih.gov/pubmed/?cmd=HistorySearch&querykey=1) |

EMBASE

**Embase Session Results (12 Dec 2019)**

| **No.** | **Query** | **Results** |
| --- | --- | --- |
| #5 | #4 AND ([article]/lim OR [article in press]/lim OR [review]/lim) AND [humans]/lim | **723** |
| #4 | #1 AND #2 AND #3 | **1071** |
| #3 | 'treatment outcome'/exp OR 'treatment response'/exp OR 'computer prediction'/exp OR 'outcome assessment':ti,ab,kw OR 'treatment outcome':ti,ab,kw OR 'tumor respons*':ti,ab,kw OR 'tumour respons*':ti,ab,kw OR 'chemo respons*':ti,ab,kw OR 'treatment respons*':ti,ab,kw OR 'therapy respons*':ti,ab,kw OR 'chemotherapy respons*':ti,ab,kw OR 'response assess*':ti,ab,kw OR 'response evaluat*':ti,ab,kw OR 'response prediction':ti,ab,kw OR 'predictive respons*':ti,ab,kw OR 'predicting respons*':ti,ab,kw OR 'outcome prediction*':ti,ab,kw OR 'predictive outcome*':ti,ab,kw OR 'predicting outcome*':ti,ab,kw | **1853431** |
| #2 | 'artificial intelligence'/exp OR 'computational intelligen*':ti,ab,kw OR 'machine learning'/exp OR 'machine intelligen*':ti,ab,kw OR 'computer reasoning':ti,ab,kw OR 'artificial intelligen*':ti,ab,kw OR 'computer vision':ti,ab,kw OR 'machine learning':ti,ab,kw OR 'fuzzy logic':ti,ab,kw OR 'decision support system'/exp OR 'computer assisted diagnosis'/de OR 'image interpretation'/exp OR 'image processing'/exp OR 'computer-assisted image interpretation*':ti,ab,kw OR 'computer-assisted radiographic image interpretation*':ti,ab,kw OR 'computer assisted diagnos*':ti,ab,kw OR 'computer-assisted decision making':ti,ab,kw OR 'automatic diagnos*':ti,ab,kw OR 'computer diagnos*':ti,ab,kw OR 'computer-assisted diagnos*':ti,ab,kw OR 'radiomics'/exp OR 'radiogenomics'/exp OR radiomic*:ti,ab,kw OR radiogenomic*:ti,ab,kw OR 'data mining':ti,ab,kw OR 'datamining':ti,ab,kw OR 'big data'/exp OR 'big data':ti,ab,kw OR 'bigdata':ti,ab,kw OR 'texture analysis'/exp OR 'texture anal*':ti,ab,kw OR 'textural anal*':ti,ab,kw OR 'data science'/exp OR 'data science*':ti,ab,kw OR 'data analytic*':ti,ab,kw OR 'data-driven science*':ti,ab,kw OR 'feature extracti*':ti,ab,kw OR 'quantitative image analy*':ti,ab,kw OR 'quantitative image feature*':ti,ab,kw | **338100** |
| #1 | 'digestive system tumor'/de OR 'digestive system cancer'/exp OR 'digestive system carcinoma'/exp OR 'digestive system neoplas*':ti,ab,kw OR 'gastrointestinal tumor'/exp OR 'gastrointestinal neoplas*':ti,ab,kw OR 'gastrointestinal carcinoma*':ti,ab,kw OR 'gastrointestinal tract cancer*':ti,ab,kw OR 'gastrointestinal cancer*':ti,ab,kw OR 'esophagus tumor'/de OR 'esophagus cancer'/exp OR 'esophageal neoplasm*':ti,ab,kw OR 'esophageal cancer*':ti,ab,kw OR 'oesophageal neoplasm*':ti,ab,kw OR 'oesophageal cancer*':ti,ab,kw OR 'esophageal carcinoma*':ti,ab,kw OR 'esophageal tumor*':ti,ab,kw OR 'esophageal tumour*':ti,ab,kw OR 'oesophageal carcinoma*':ti,ab,kw OR 'oesophageal tumor*':ti,ab,kw OR 'oesophageal tumour*':ti,ab,kw OR 'stomach tumor'/de OR 'stomach neoplasm*':ti,ab,kw OR 'stomach cancer'/exp OR 'gastric neoplasm*':ti,ab,kw OR 'cancer of stomach':ti,ab,kw OR 'stomach cancer*':ti,ab,kw OR 'gastric cancer*':ti,ab,kw OR 'cancer of the stomach':ti,ab,kw OR 'gastric carcinoma*':ti,ab,kw OR 'gastric tumor*':ti,ab,kw OR 'gastric tumour*':ti,ab,kw OR 'intestine cancer'/exp OR 'intestinal neoplasm*':ti,ab,kw OR 'intestines neoplasm*':ti,ab,kw OR 'cancer of intestine*':ti,ab,kw OR 'intestines cancer*':ti,ab,kw OR 'cancer of the intestine*':ti,ab,kw OR 'intestinal cancer*':ti,ab,kw OR 'small bowel cancer*':ti,ab,kw OR 'duodenal neoplasm*':ti,ab,kw OR 'duodenum neoplasm*':ti,ab,kw OR 'duodenal cancer*':ti,ab,kw OR 'cancer of the duodenum':ti,ab,kw OR 'cancer of duodenum':ti,ab,kw OR 'duodenum cancer*':ti,ab,kw OR 'duodenum carcinoma*':ti,ab,kw OR 'duodenal carcinoma*':ti,ab,kw OR 'duodenum tumor*':ti,ab,kw OR 'duodenal tumor*':ti,ab,kw OR 'duodenum tumour*':ti,ab,kw OR 'duodenal tumour*':ti,ab,kw OR 'duodenal metastasis':ti,ab,kw OR 'jejunal cancer*':ti,ab,kw OR 'jejunal neoplasm*':ti,ab,kw OR 'jejunal carcinoma*':ti,ab,kw OR 'jejunal tumor*':ti,ab,kw OR 'jejunal tumour*':ti,ab,kw OR 'jejunal metastas*':ti,ab,kw OR 'ileal cancer*':ti,ab,kw OR 'ileal neoplasm*':ti,ab,kw OR 'ileal carcinoma*':ti,ab,kw OR 'ileal tumor*':ti,ab,kw OR 'ileal tumour*':ti,ab,kw OR 'ileal metastas*':ti,ab,kw OR 'ileocecal cancer*':ti,ab,kw OR 'ileocecal carcinoma*':ti,ab,kw OR 'ileocecal tumor*':ti,ab,kw OR 'cecal cancer*':ti,ab,kw OR 'cecal neoplasm*':ti,ab,kw OR 'cecal carcinoma*':ti,ab,kw OR 'cecal tumor*':ti,ab,kw OR 'peritoneal metastas*':ti,ab,kw OR 'colorectal tumor'/de OR 'colonic neoplas*':ti,ab,kw OR 'colon neoplas*':ti,ab,kw OR 'cancer of colon':ti,ab,kw OR 'cancer of the colon':ti,ab,kw OR 'colon cancer*':ti,ab,kw OR 'colonic cancer*':ti,ab,kw OR 'colon tumour*':ti,ab,kw OR 'colon tumor*':ti,ab,kw OR 'colonic tumour*':ti,ab,kw OR 'colonic tumor*':ti,ab,kw OR 'colorectal neoplas*':ti,ab,kw OR 'colorectal tumor*':ti,ab,kw OR 'colorectal tumour*':ti,ab,kw OR 'colorectal carcinoma*':ti,ab,kw OR 'colorectal cancer*':ti,ab,kw OR 'colorectal metastas*':ti,ab,kw OR 'colorectal lung metastas*':ti,ab,kw OR 'metastatic colorectal*':ti,ab,kw OR 'rectal cancer*':ti,ab,kw OR 'rectal neoplasm*':ti,ab,kw OR 'rectal tumor*':ti,ab,kw OR 'rectal tumour*':ti,ab,kw OR 'rectal metastas*':ti,ab,kw OR 'rectal carcinoma*':ti,ab,kw OR 'pancreas tumor'/de OR 'pancreas cancer'/exp OR 'pancreatic neoplasm*':ti,ab,kw OR 'pancreas neoplasm*':ti,ab,kw OR 'cancer of pancreas':ti,ab,kw OR 'pancreas cancer*':ti,ab,kw OR 'pancreatic cancer*':ti,ab,kw OR 'cancer of the pancreas':ti,ab,kw OR 'pancreas tumor*':ti,ab,kw OR 'pancreas tumour*':ti,ab,kw OR 'pancreas metastas*':ti,ab,kw OR 'pancreas carcinoma*':ti,ab,kw OR 'pancreatic tumor*':ti,ab,kw OR 'pancreatic tumour*':ti,ab,kw OR 'pancreatic metastas*':ti,ab,kw OR 'pancreatic carcinoma*':ti,ab,kw OR 'liver tumor'/de OR 'hepatobiliary system cancer'/exp OR 'liver neoplas*':ti,ab,kw OR 'hepatic neoplasm*':ti,ab,kw OR 'cancer of liver':ti,ab,kw OR 'cancer of the liver':ti,ab,kw OR 'hepatic cancer*':ti,ab,kw OR 'liver cancer*':ti,ab,kw OR 'liver cell adenoma*':ti,ab,kw OR 'liver cell carcinoma*':ti,ab,kw OR 'liver carcinoma*':ti,ab,kw OR 'liver cell cancer*':ti,ab,kw OR 'liver cell tumor*':ti,ab,kw OR 'liver cell tumour*':ti,ab,kw OR 'liver metastas*':ti,ab,kw OR 'hepatic metastas*':ti,ab,kw OR 'hepatic colorectal metastas*':ti,ab,kw OR 'colorectal liver metastas*':ti,ab,kw OR 'liver tumor*':ti,ab,kw OR 'liver tumour*':ti,ab,kw OR 'biliary tract neoplas*':ti,ab,kw OR 'gallbladder neoplas*':ti,ab,kw OR 'gallbladder cancer*':ti,ab,kw OR 'gallbladder metastas*':ti,ab,kw OR 'gallbladder tumor*':ti,ab,kw OR 'gallbladder tumour*':ti,ab,kw OR 'gallbladder carcinoma*':ti,ab,kw OR 'bile duct neoplas*':ti,ab,kw OR 'bile duct cancer*':ti,ab,kw OR 'cancer of the bile duct':ti,ab,kw OR 'cancer of bile duct':ti,ab,kw OR 'bile duct carcinoma*':ti,ab,kw OR 'gastrointestinal stromal tumor'/exp OR 'gastrointestinal stromal tumor*':ti,ab,kw OR 'gastrointestinal stromal tumour*':ti,ab,kw OR 'gastrointestinal stromal neoplasm*':ti,ab,kw OR 'gastrointestinal stromal cancer*':ti,ab,kw OR 'gastrointestinal stromal sarcoma*':ti,ab,kw OR 'gastrointestinal neuroendocrine tumor'/exp OR 'gastroenteropancreatic neuroendocrine tumor'/exp OR 'gastrointestinal neuroendocrine neoplasm*':ti,ab,kw OR 'gastrointestinal neuroendocrine tumor*':ti,ab,kw OR 'gastrointestinal neuroendocrine tumour*':ti,ab,kw OR 'gastrointestinal neuroendocrine carcinoma*':ti,ab,kw OR 'pancreatic neuroendocrine tumor*':ti,ab,kw OR 'pancreatic neuroendocrine tumour*':ti,ab,kw OR 'pancreatic neuroendocrine carcinoma*':ti,ab,kw OR 'pancreatic neuroendocrine neoplasm*':ti,ab,kw OR 'gastric neuroendocrine tumor*':ti,ab,kw OR 'gastric neuroendocrine tumour*':ti,ab,kw OR 'gastric neuroendocrine carcinoma*':ti,ab,kw OR 'gastric neuroendocrine neoplasm*':ti,ab,kw OR 'intestinal neuroendocrine tumor*':ti,ab,kw OR 'intestinal neuroendocrine neoplasm*':ti,ab,kw OR 'esophageal neuroendocrine tumor*':ti,ab,kw OR 'esophageal neuroendocrine carcinoma*':ti,ab,kw OR 'esophageal neuroendocrine neoplasm*':ti,ab,kw OR 'rectal neuroendocrine tumor*':ti,ab,kw OR 'rectal neuroendocrine tumour*':ti,ab,kw OR 'rectal neuroendocrine carcinoma*':ti,ab,kw OR 'rectal neuroendocrine neoplasm*':ti,ab,kw OR 'hepatic neuroendocrine tumor*':ti,ab,kw OR 'hepatic neuroendocrine tumour*':ti,ab,kw OR 'hepatic neuroendocrine carcinoma*':ti,ab,kw OR 'hepatic neuroendocrine neoplasm*':ti,ab,kw | **1005793** |

## Cochrane Library

Search name

Date Run: 12/12/2019 11:17:25

Comment:

**ID Search Hits**

#1 Digestive NEXT System NEXT Neoplas*:ti,ab,kw OR Gastrointestinal NEXT Neoplas*:ti,ab,kw OR gastrointestinal NEXT carcinoma*:ti,ab,kw OR Gastrointestinal NEXT Tract NEXT Cancer*:ti,ab,kw OR Gastrointestinal NEXT Cancer*:ti,ab,kw OR Esophageal NEXT Neoplasm*:ti,ab,kw OR Esophageal NEXT cancer*:ti,ab,kw OR Oesophageal NEXT Neoplasm*:ti,ab,kw OR Oesophageal NEXT cancer*:ti,ab,kw OR Esophageal NEXT carcinoma*:ti,ab,kw OR Esophageal NEXT tumor*:ti,ab,kw OR Esophageal NEXT tumour*:ti,ab,kw OR Oesophageal NEXT carcinoma*:ti,ab,kw OR Oesophageal NEXT tumor*:ti,ab,kw OR Oesophageal NEXT tumour*:ti,ab,kw OR Stomach NEXT Neoplasm*:ti,ab,kw OR Gastric NEXT Neoplasm*:ti,ab,kw OR Cancer NEXT of NEXT Stomach:ti,ab,kw OR Stomach NEXT Cancer*:ti,ab,kw OR Gastric NEXT Cancer*:ti,ab,kw OR Cancer NEXT of NEXT the NEXT Stomach:ti,ab,kw OR gastric NEXT carcinoma*:ti,ab,kw OR gastric NEXT tumor*:ti,ab,kw OR gastric NEXT tumour*:ti,ab,kw OR Intestinal NEXT Neoplasm*:ti,ab,kw OR Intestines NEXT Neoplasm*:ti,ab,kw OR Cancer NEXT of NEXT Intestine*:ti,ab,kw OR Intestines NEXT Cancer*:ti,ab,kw OR Cancer NEXT of NEXT the NEXT Intestine*:ti,ab,kw OR Intestinal NEXT Cancer*:ti,ab,kw OR small NEXT bowel NEXT cancer*:ti,ab,kw OR Duodenal NEXT Neoplasm*:ti,ab,kw OR duodenum NEXT neoplasm*:ti,ab,kw OR Duodenal NEXT Cancer*:ti,ab,kw OR Cancer NEXT of NEXT the NEXT Duodenum:ti,ab,kw OR Cancer NEXT of Duodenum:ti,ab,kw OR Duodenum NEXT Cancer*:ti,ab,kw OR duodenum NEXT carcinoma*:ti,ab,kw OR duodenal NEXT carcinoma*:ti,ab,kw OR duodenum NEXT tumor*:ti,ab,kw OR duodenal NEXT tumor*:ti,ab,kw OR duodenum NEXT tumour*:ti,ab,kw OR duodenal NEXT tumour*:ti,ab,kw OR "duodenal metastasis":ti,ab,kw OR jejunal NEXT cancer*:ti,ab,kw OR jejunal NEXT neoplasm*:ti,ab,kw OR jejunal NEXT carcinoma*:ti,ab,kw OR jejunal NEXT tumor*:ti,ab,kw OR jejunal NEXT tumour*:ti,ab,kw OR jejunal NEXT metastas*:ti,ab,kw OR ileal NEXT cancer*:ti,ab,kw OR ileal NEXT neoplasm*:ti,ab,kw OR ileal NEXT carcinoma*:ti,ab,kw OR ileal NEXT tumor*:ti,ab,kw OR ileal NEXT tumour*:ti,ab,kw OR ileal NEXT metastas*:ti,ab,kw OR ileocecal NEXT cancer*:ti,ab,kw OR ileocecal NEXT carcinoma*:ti,ab,kw OR ileocecal NEXT tumor*:ti,ab,kw OR cecal NEXT cancer*:ti,ab,kw OR cecal NEXT neoplasm*:ti,ab,kw OR cecal NEXT carcinoma*:ti,ab,kw OR cecal NEXT tumor*:ti,ab,kw OR peritoneal NEXT metastas*:ti,ab,kw OR Colonic NEXT Neoplas*:ti,ab,kw OR Colon NEXT Neoplas*:ti,ab,kw OR "Cancer of Colon":ti,ab,kw OR "Cancer of the Colon":ti,ab,kw OR Colon NEXT Cancer*:ti,ab,kw OR Colonic NEXT Cancer*:ti,ab,kw OR Colon NEXT tumour*:ti,ab,kw OR colon NEXT tumor*:ti,ab,kw OR colonic NEXT tumour*:ti,ab,kw OR colonic NEXT tumor*:ti,ab,kw OR Colorectal NEXT Neoplas*:ti,ab,kw OR Colorectal NEXT Tumor*:ti,ab,kw OR Colorectal NEXT Tumour*:ti,ab,kw OR Colorectal NEXT Carcinoma*:ti,ab,kw OR Colorectal NEXT Cancer*:ti,ab,kw OR colorectal NEXT metastas*:ti,ab,kw OR colorectal NEXT lung NEXT metastas*:ti,ab,kw OR metastatic NEXT colorectal*:ti,ab,kw OR rectal NEXT cancer*:ti,ab,kw OR rectal NEXT neoplasm*:ti,ab,kw OR rectal NEXT tumor*:ti,ab,kw OR rectal NEXT tumour*:ti,ab,kw OR rectal NEXT metastas*:ti,ab,kw OR rectal NEXT carcinoma*:ti,ab,kw OR Pancreatic NEXT Neoplasm*:ti,ab,kw OR Pancreas NEXT Neoplasm*:ti,ab,kw OR "Cancer of Pancreas":ti,ab,kw OR Pancreas NEXT Cancer*:ti,ab,kw OR Pancreatic NEXT Cancer*:ti,ab,kw OR "Cancer of the Pancreas":ti,ab,kw OR pancreas NEXT tumor*:ti,ab,kw OR pancreas NEXT tumour*:ti,ab,kw OR pancreas NEXT metastas*:ti,ab,kw OR pancreas NEXT carcinoma*:ti,ab,kw OR pancreatic NEXT tumor*:ti,ab,kw OR pancreatic NEXT tumour*:ti,ab,kw OR pancreatic NEXT metastas*:ti,ab,kw OR pancreatic NEXT carcinoma*:ti,ab,kw OR Liver NEXT Neoplas*:ti,ab,kw OR Hepatic NEXT Neoplasm*:ti,ab,kw OR "Cancer of Liver":ti,ab,kw OR "Cancer of the Liver":ti,ab,kw OR Hepatic NEXT Cancer*:ti,ab,kw OR Liver NEXT Cancer*:ti,ab,kw OR Liver NEXT Cell NEXT Adenoma*:ti,ab,kw OR liver NEXT cell NEXT carcinoma*:ti,ab,kw OR liver NEXT carcinoma*:ti,ab,kw OR liver NEXT cell NEXT cancer*:ti,ab,kw OR liver NEXT cell NEXT tumor*:ti,ab,kw OR liver NEXT cell NEXT tumour*:ti,ab,kw OR liver NEXT metastas*:ti,ab,kw OR hepatic NEXT metastas*:ti,ab,kw OR hepatic NEXT colorectal NEXT metastas*:ti,ab,kw OR colorectal NEXT liver NEXT metastas*:ti,ab,kw OR liver NEXT tumor*:ti,ab,kw OR liver NEXT tumour*:ti,ab,kw OR Biliary NEXT Tract NEXT Neoplas*:ti,ab,kw OR gallbladder NEXT neoplas*:ti,ab,kw OR gallbladder NEXT cancer*:ti,ab,kw OR gallbladder NEXT metastas*:ti,ab,kw OR gallbladder NEXT tumor*:ti,ab,kw OR gallbladder NEXT tumour*:ti,ab,kw OR gallbladder NEXT carcinoma*:ti,ab,kw OR bile NEXT duct NEXT neoplas*:ti,ab,kw OR bile NEXT duct NEXT cancer*:ti,ab,kw OR "Cancer of the Bile Duct":ti,ab,kw OR "Cancer of Bile Duct":ti,ab,kw OR bile NEXT duct NEXT carcinoma*:ti,ab,kw OR Gastrointestinal NEXT Stromal NEXT Tumor*:ti,ab,kw OR Gastrointestinal NEXT Stromal NEXT Tumour*:ti,ab,kw OR Gastrointestinal NEXT Stromal NEXT Neoplasm*:ti,ab,kw OR Gastrointestinal NEXT Stromal NEXT Cancer*:ti,ab,kw OR Gastrointestinal NEXT stromal NEXT sarcoma*:ti,ab,kw OR Gastrointestinal NEXT neuroendocrine NEXT neoplasm*:ti,ab,kw OR Gastrointestinal NEXT neuroendocrine NEXT tumor*:ti,ab,kw OR Gastrointestinal NEXT neuroendocrine NEXT tumour*:ti,ab,kw OR Gastrointestinal NEXT neuroendocrine NEXT carcinoma*:ti,ab,kw OR pancreatic NEXT neuroendocrine NEXT tumor*:ti,ab,kw OR pancreatic NEXT neuroendocrine NEXT tumour*:ti,ab,kw OR pancreatic NEXT neuroendocrine NEXT carcinoma*:ti,ab,kw OR pancreatic NEXT neuroendocrine NEXT neoplasm*:ti,ab,kw OR gastric NEXT neuroendocrine NEXT tumor*:ti,ab,kw OR gastric NEXT neuroendocrine NEXT tumour*:ti,ab,kw OR gastric NEXT neuroendocrine NEXT carcinoma*:ti,ab,kw OR gastric NEXT neuroendocrine NEXT neoplasm*:ti,ab,kw OR intestinal NEXT neuroendocrine NEXT tumor*:ti,ab,kw OR intestinal NEXT neuroendocrine NEXT tumor*:ti,ab,kw OR intestinal NEXT neuroendocrine NEXT neoplasm*:ti,ab,kw OR esophageal NEXT neuroendocrine NEXT tumor*:ti,ab,kw OR esophageal NEXT neuroendocrine NEXT carcinoma*:ti,ab,kw OR esophageal NEXT neuroendocrine NEXT neoplasm*:ti,ab,kw OR rectal NEXT neuroendocrine NEXT tumor*:ti,ab,kw OR rectal NEXT neuroendocrine NEXT tumour*:ti,ab,kw OR rectal NEXT neuroendocrine NEXT carcinoma*:ti,ab,kw OR rectal NEXT neuroendocrine NEXT neoplasm*:ti,ab,kw OR hepatic NEXT neuroendocrine NEXT tumor*:ti,ab,kw OR hepatic NEXT neuroendocrine NEXT tumour*:ti,ab,kw OR hepatic NEXT neuroendocrine NEXT carcinoma*:ti,ab,kw OR hepatic NEXT neuroendocrine NEXT neoplasm*:ti,ab,kw **44264**

#2 Computational NEXT Intelligen*:ti,ab,kw OR Machine NEXT Intelligen*:ti,ab,kw OR "Computer Reasoning":ti,ab,kw OR Artificial NEXT Intelligen*:ti,ab,kw OR "Computer Vision":ti,ab,kw OR "Machine learning":ti,ab,kw OR "fuzzy logic":ti,ab,kw OR Computer NEXT Assisted NEXT Image NEXT Interpretation*:ti,ab,kw OR Computer NEXT Assisted NEXT Radiographic NEXT Image NEXT Interpretation*:ti,ab,kw OR computer NEXT assisted NEXT diagnos*:ti,ab,kw OR "computer assisted decision making":ti,ab,kw OR automatic NEXT diagnos*:ti,ab,kw OR computer NEXT diagnos*:ti,ab,kw OR computer NEXT assisted NEXT diagnos*:ti,ab,kw OR radiomic*:ti,ab,kw OR radiogenomic*:ti,ab,kw OR "Data Mining":ti,ab,kw OR "Datamining":ti,ab,kw OR "Big Data":ti,ab,kw OR "bigdata":ti,ab,kw OR texture NEXT anal*:ti,ab,kw OR textural NEXT anal*:ti,ab,kw OR Data NEXT Science*:ti,ab,kw OR Data NEXT Analytic*:ti,ab,kw OR Data NEXT Driven NEXT Science*:ti,ab,kw OR feature NEXT extracti*:ti,ab,kw OR quantitative NEXT image NEXT analy*:ti,ab,kw OR quantitative NEXT image NEXT feature*:ti,ab,kw **2294**

#3 "Outcome Assessment":ti,ab,kw OR "treatment outcome":ti,ab,kw OR Tumor NEXT respons*:ti,ab,kw OR tumour respons*:ti,ab,kw OR chemo respons*:ti,ab,kw OR treatment NEXT respons*:ti,ab,kw OR therapy NEXT respons*:ti,ab,kw OR chemotherapy NEXT respons*:ti,ab,kw OR response NEXT assess*:ti,ab,kw OR response NEXT evaluat*:ti,ab,kw OR "response prediction":ti,ab,kw OR predictive NEXT respons*:ti,ab,kw OR predicting NEXT respons*:ti,ab,kw OR outcome NEXT prediction*:ti,ab,kw OR predictive NEXT outcome*:ti,ab,kw OR predicting NEXT outcome*:ti,ab,kw **249012**

#4 #1 AND #2 AND #3 in Cochrane Reviews, Trials **33**
